# Reconstructing the ischemic osteogenic microenvironment through hierarchical scaffolds orchestrating Mg^2+^ signaling and neuropilin-1–mediated angiogenesis

**DOI:** 10.1016/j.bioactmat.2026.02.031

**Published:** 2026-03-26

**Authors:** Zijie Pei, Haojing Xu, Piqian Zhao, Ya Wen, Ze Zhang, Liangkun Huang, Mengyu Wang, Bo Peng, Liangyuan Wen, Peng Wen, Fengpo Sun

**Affiliations:** a Department of Orthopedics, Beijing Hospital, National Center of Gerontology, Institute of Geriatric Medicine, Chinese Academy of Medical Sciences, China; b State Key Laboratory of Clean and Efficient Turbomachinery Power Equipment, Department of Mechanical Engineering, Tsinghua University, China; c Peking Union Medical College, China; d Department of Orthopedics, The Fourth Affiliated Hospital of School of Medicine, and International School of Medicine, International Institutes of Medicine, Zhejiang University, China; e Capital Medical University School of Biomedical Engineering, China

**Keywords:** Ischemic bone defect, Magnesium scaffold, Neuropilin-1, Angiogenesis, Osteogenesis

## Abstract

Ischemic bone defects remain a major challenge in bone tissue engineering, primarily due to insufficient angiogenesis caused by severely compromised local blood supply. Here, we develop a bioactive implant that mechanistically amplifies magnesium-induced angiogenesis by integrating pro-angiogenic liposomes encapsulating neuropilin-1 (NRP-1) with a 3D-printed magnesium (Mg) alloy porous scaffold. The customized Mg scaffold matched defect morphology, provided reliable mechanical support, and featured interconnected micropores to facilitate bone ingrowth and integration. Composite surface coatings simultaneously moderated the rapid degradation of Mg and supplied abundant binding sites for NRP-1-loaded lipos, enabling coordinated regulation of angiogenesis and osteogenesis. In an ischemic bone defect model, the scaffold significantly enhanced neovascularization, bone formation density, and interfacial osseointegration. Mechanistically, the controlled release of Mg^2+^ promoted osteogenic differentiation and upregulated Vascular Endothelial Growth Factor A (VEGFA) expression, establishing a biochemical foundation for angiogenesis. Importantly, the incorporation of NRP-1 further potentiated VEGFA–VEGFR2 signaling, markedly amplifying angiogenic efficacy. Collectively, this work identifies an Mg/NRP-1-mediated coupling axis of osteogenesis and angiogenesis, providing a mechanistically informed strategy for bone regeneration in severely ischemic microenvironments.

## Highlights

• A hierarchically constructed Mg-based scaffold enables coupled osteogenesis and angiogenesis under ischemic conditions.
• Composite HTO–LDH–PDA coatings precisely regulate Mg degradation and support bioactive molecule loading.
• Liposome-encapsulated Neuropilin-1 (Lipo@NRP-1) synergizes with Mg^2+^ release to amplify VEGFA–VEGFR2 signaling.
• The Mg/NRP-1-mediated coupling axis promotes integrated bone–vascular regeneration in ischemic defects.

## Graphical abstract

**Figure:**
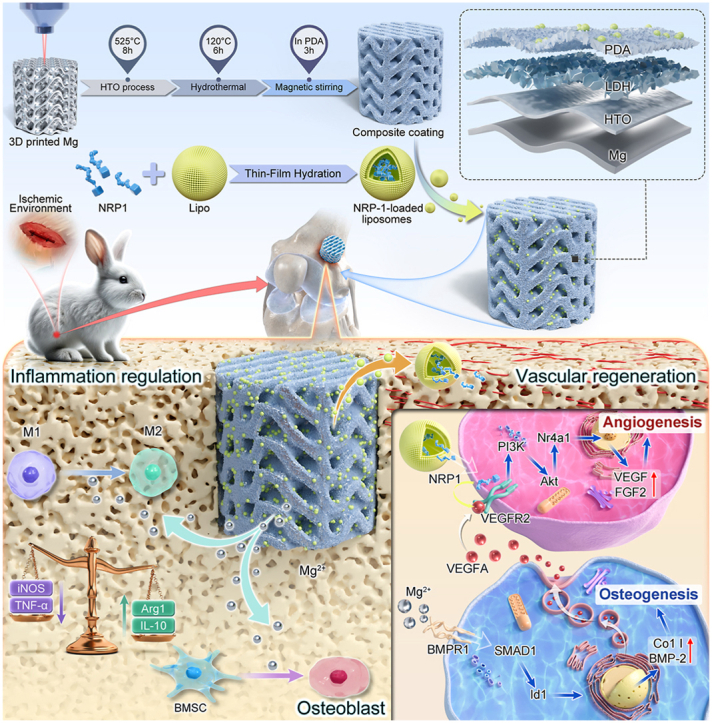

## 1 Introduction

The treatment of ischemic bone defects remains a significant clinical challenge, as it requires the simultaneously restoration of vascular function and regeneration of bone tissue in a compromised microenvironment [1]. This dual obstacle contributes to poor bone healing outcomes in conditions such as delayed union or nonunion of distal tibial fractures, diabetic foot, and steroid-induced femoral head necrosis. According to the Association Research Circulation Osseous (ARCO), osteonecrosis is defined as a complex pathological process involving the interruption of intraosseous blood flow caused by mechanical, biological, or metabolic factors, leading to cell death and dysregulated bone metabolism [2]. Addressing these problems holds significant clinical value, particularly in the context of an aging population and increasing incidence of metabolic and traumatic bone disorders, characterized by impaired bone regeneration secondary to localized vascular insufficiency, which fundamentally disrupts the bone healing process [3,4]. Within this pathological microenvironment, the functional reconstruction of the bone microvasculature plays a critical role in bone healing, not only by providing essential metabolic support but also by regulating osteogenesis, bone remodeling, and inflammation [5]. However, conventional bone repair strategies that primarily focus on "bone filling", typically including autologous or allogeneic bone grafts, vascularized bone flaps, the Masquelet induced membrane technique, and distraction osteogenesis for large-segment bone defects, often neglect the necessity of revascularization, resulting in delayed or failed bone regeneration and subsequent complications such as nonunion or delayed union under ischemic conditions [6,7]. Therefore, developing strategies that not only concurrently promote osteogenesis and restore the local vascular network, but also mechanistically enhance angiogenic signaling within the ischemic microenvironment, has become a pressing scientific and clinical challenge in the treatment of ischemic bone defects.

Mg and its alloys have emerged as a promising biomaterial for orthopedic applications due to its cytocompatibility, biodegradability, and osteogenic properties [[8], [9], [10], [11]]. Mg is a life element, and Mg^2+^ have been proved to modulate the immune microenvironment by promoting macrophage polarization toward a pro-regenerative phenotype, thereby contributing to a more favorable environment for tissue repair [12]. For instance, as a rare earth-containing Mg alloy with superior strength and cytocompatibility, WE43 has been among the rare biodegradable metals that are approved in clinical applications including orthopedic fixture and vascular intervention [13,14]. The advent of additive manufacturing technologies, especially laser powder bed fusion (LPBF), has enabled the precise fabrication of WE43 porous scaffolds with anatomical morphology and tailored architectures, which not only can provide reliable mechanical support and enhanced stress stimulation [15,16], but also can offer favorable conditions for cellular infiltration, nutrient diffusion, and vascular ingrowth [17,18], both essential for effective bone regeneration and integration.

Despite these advances, a major limitation of Mg alloy porous scaffolds fabricated by LPBF remains the excessively rapid degradation in physiological environments, which can be attributed to the intrinsic mechanism of Mg corrosion, the widespread distribution of secondary phases and the enlarged surface area, and consequently can lead to problems such as excessive release of hydrogen, local ion enrichment, and premature loss of structural integrity [19,20]. Liu et al. reported the structural integrity of as-printed WE43 scaffolds was seriously damaged after 12 h by in vitro immersion test in Hank's solution and after 4 weeks by implantation test in the rabbits' femur [21]. Although surface coating can protect Mg alloys from rapid degradation, conventional approaches are difficult to achieve uniform coverage and stable adhesion in porous scaffolds [22,23]. High-temperature oxidation (HTO) treatment enables the in-situ formation of a dense and stable rare earth oxide layer on the surface of WE43 scaffold, offering effective protection for the underlying delicate porous structure without introducing extra elements [24]. By further constructing layered double hydroxides (LDH) above the HTO layer, the bone regeneration of scaffolds was significantly improved due to the tempered degradation and surface nano-textures [25]. Meanwhile, the regulated release of Mg^2+^ was found to upregulate the expression of VEGFA and induce endothelial cell differentiation, thereby promoting angiogenic responses [25,26].

Vascularization plays a fundamental role in bone regeneration by delivering oxygen and nutrients, removing metabolic waste, and providing paracrine signals that regulate osteogenic differentiation, bone remodeling, and immune modulation [27]. In ischemic bone defects, the absence of a functional vascular network severely disrupts the local microenvironment and remains a major barrier to bone repair. Mg alloy scaffolds have shown promise in promoting angiogenesis and osteogenesis; however, their ability to induce efficient vascular reconstruction and robust bone regeneration remains limited in ischemic bone defects, where angiogenesis driven solely by endogenous VEGFA upregulation lacks sufficient signaling efficiency and downstream activation. Consequently, there is an urgent need for more targeted regulatory approaches that enhance VEGFA signaling efficiency through ligand-mediated modulation, thereby activating downstream angiogenic pathways and promoting the formation of functional vascular networks. Recent efforts have focused on developing functionally enhanced bone repair materials incorporating bioactive proteins and growth factors [28,29]. These advanced strategies enhance osteo-vascular coupling by precisely modulating key angiogenic signaling nodes to coordinate angiogenic and osteogenic pathways. Their high specificity, sustained bioactivity, and ability to coordinate multiple regenerative processes make them a powerful and promising direction for the treatment of ischemic bone defects. We further hypothesized that, although magnesium ions can upregulate VEGFA expression, insufficient VEGFA–VEGFR2 signaling efficiency represents a critical bottleneck in ischemic angiogenesis, and that NRP-1 functions as a key regulatory node to amplify VEGFA signaling rather than merely increasing VEGFA availability.

NRP-1 is a non-tyrosine kinase co-receptor of VEGFA that plays a pivotal role in angiogenesis by regulating the efficiency and specificity of VEGF signaling. Mechanistically, NRP-1 forms a stable ternary complex with VEGFR2 to enhance VEGFA binding and signal transduction, thereby acting as a critical amplifier of downstream angiogenic responses [30]. In addition to its role in promoting endothelial cell migration, proliferation, and lumen formation, NRP-1 has been implicated in vascular sprouting and stabilization during neovascularization [31,32]. These properties make NRP-1 an attractive therapeutic molecule for enhancing vascular regeneration, particularly in ischemic bone defects where angiogenic responses are critically impaired. However, the practical application of NRP-1 in biomaterial systems is hindered by its intrinsic protein properties such as structural instability, susceptibility to enzymatic degradation, and weak affinity for scaffold surfaces, making it difficult to achieve sustained bioactivity at the implantation site. Effective immobilization and controlled release of NRP-1 at the scaffold–tissue interface are therefore essential to maximize its therapeutic efficacy.

**Fig. 1:**
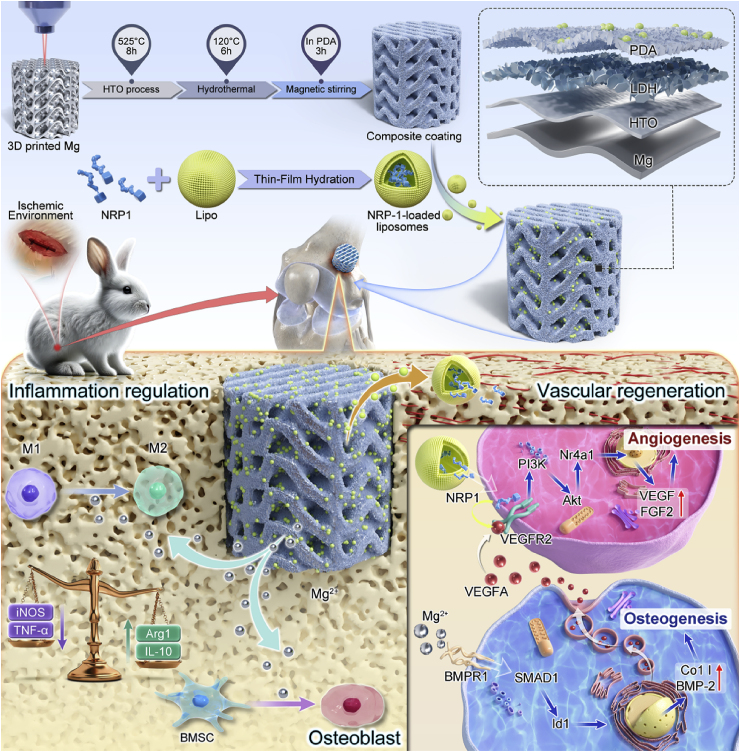
Schematic illustration of the design, functionalization, and mechanism of a hierarchically constructed porous scaffold for ischemic bone defect repair. The Mg alloy scaffold was fabricated by 3D printing and sequentially modified with HTO–LDH–PDA composite coatings to regulate degradation and enhance interfacial bioactivity. NRP-1-loaded lipos were immobilized onto the PDA layer to achieve sustained local release. In vivo, the scaffold facilitates macrophage polarization, modulates the inflammatory microenvironment, and promotes osteogenic differentiation. Meanwhile, Mg^2+^ enhances VEGFA expression, and NRP-1 synergistically amplifies VEGFA/VEGFR2 signaling, promoting angiogenesis and coupling with osteogenesis to support coordinated bone regeneration under ischemic conditions.

We hypothesized that, in ischemic bone defects, reinforcing VEGFA signaling efficiency through NRP-1–mediated modulation on a magnesium-based scaffold could restore the coupling between angiogenesis and osteogenesis, thereby promoting functional vascular reconstruction and bone regeneration. In this study, we aimed to address the critical challenge of impaired coupling of angiogenesis and osteogenesis in ischemic bone defects by mechanistically reinforcing angiogenic signaling through the construction of a multifunctional composite scaffold integrating Mg alloy and NRP-1 (Fig. 1). Mg alloy porous scaffolds were designed by the triply periodic minimal surface approach and printed by LPBF process using WE43 powder. The customized scaffold provided mechanical support and osteogenic potential as the bone grafting base. HTO and LDH layers were coated in sequence on the surface of WE43 scaffold to regulate the degradation rate and the release of Mg ions, as well as increase surface area for further functionalization with NRP-1 protein. The NRP-1 was encapsulated in liposomes (Lipo), which acted as nanocarriers due to their excellent biocompatibility and phospholipid bilayer structure to load the bioactive protein [[33], [34], [35]]. In addition, inspired by the adhesive proteins of mussels, polydopamine (PDA) was introduced onto the surface of HTO-LDH layer in order to establish multiple types of interactions, such as π–π stacking, hydrogen bonding, and covalent bonding, with the phospholipid bilayers of liposomes [36,37]. The HTO-LDH-PDA layer was purposed to serve as a versatile interface for the immobilization of NRP-1-loaded lipos, enabling localized and sustained delivery of pro-angiogenic signals. Overall, anchored in the pathological features and regenerative demands of ischemic bone defects, this composite scaffold systematically integrates structural, chemical, and biological cues in hierarchical design, and is expected to orchestrate osteogenic, angiogenic, and immunomodulatory responses in a coordinated manner, offering a rational and potentially effective approach toward precision therapy for ischemic bone regeneration.

## 2 Materials and methods

### 2.1 Fabrication of porous Mg scaffolds and composite coatings

Spherical WE43 powder with a particle size range of 20–63 μm was utilized to fabricate Mg scaffolds. The powder composition included 3.66% Y, 1.09% Gd, 2.09% Nd, 0.40% Zr, 0.01% Mn, and 0.20% Zn, with the remainder being Mg (92.55%). Porous WE43 scaffolds, each with external dimensions of Φ5 × 5 mm^3^ and a target porosity of 60%, were produced using a laser powder bed fusion (L-PBF) system (BLT S210, Xi'an, China), equipped with a 70 μm laser spot diameter. Prior to the printing process, the print substrate was preheated to 200 °C. To achieve high-quality scaffold fabrication, the process was conducted with a laser power of 80 W, a scanning speed of 800 mm/s, a layer thickness of 20 μm, and a hatch spacing of 70 μm. Laser scanning followed a zig-zag exposure strategy, with a 67° rotation between successive layers.

Following additive manufacturing, residual powder particles adhered to the surface of the WE43 Mg scaffolds. It not only potentially impacts their physical and chemical properties, but interferes with subsequent coating processes. To eliminate these particles, a chemical etching treatment was performed. Scaffolds were immersed and stirred for 120 s at room temperature in an ethanol (C_2_H_5_OH) solution containing 5% nitric acid and 5% hydrochloric acid. After etching, they were ultrasonically cleaned in pure ethanol, resulting in printed scaffolds with a silver-white metallic luster. To further modify the surface, a HTO process was applied using a muffle furnace. The scaffolds were heated at a rate of 10 °C/min to 525 °C, maintained at this temperature for 8 h, and then rapidly quenched in water at room temperature. This treatment resulted in the formation of a dense rare earth oxide layer on the surface, and the treated samples were called HTO scaffolds.

LDH coatings were synthesized on the HTO scaffolds via a hydrothermal method. Prior to the coating process, the scaffolds underwent ultrasonic cleaning in deionized water and absolute ethanol for 10 min each, followed by drying to eliminate any remaining surface contaminants. For the hydrothermal reaction, 6 ml of 1 M NaOH solution, 0.001 mol of Al(NO_3_)_3_, and 0.0005 mol of Na_2_CO_3_ were dissolved in 44 ml of deionized water. The cleaned scaffold and the prepared solution were then sealed in a high-pressure reactor, ensuring no contact between the scaffolds. The mixture was heated at 120 °C for 6 h. Upon completion, the scaffolds were cleaned again with deionized water and ethanol for 10 min and subsequently dried. The resulting samples were labeled as HTO-LDH scaffolds.

To preserve the integrity of the LDH layer during dopamine (DA) polymerization, the pH of the PDA solution was carefully adjusted. NaOH was added dropwise to a 10 mM Tris buffer (initial pH = 8.5) to increase the overall pH to 10.5, with the final volume adjusted to 100 mL. Subsequently, 1.5 mg of dopamine was dissolved in the solution to prepare the base solution. The HTO-LDH scaffolds were then immersed in the prepared solution and magnetically stirred for 3 h. During the reaction, nitrogen gas was continuously introduced at a flow rate of 50 mL/min to moderate the polymerization rate of dopamine at the elevated pH. After drying, the resulting sample was referred to as the HTO-LDH-PDA scaffold.

### 2.2 Materials characterization of Mg scaffolds

Field-emission scanning electron microscopy (FESEM, Zeiss Gemini-300, Germany) was employed to observe the scaffold morphology at an accelerating voltage of 5 kV, while energy-dispersive X-ray spectroscopy (EDX) was performed at 15 kV to analyze the elemental composition. The surface morphology and roughness of the scaffolds were characterized using AFM (Bruker Dimension Icon, USA) operated in tapping mode with a surface area of 5 μm × 5 μm. Wettability measurements were conducted with a contact angle goniometer (Data Physics OCA 20, Germany) to evaluate the hydrophilicity of the scaffold surfaces. The chemical compositions of the surface coatings were further investigated using Fourier Transform Infrared Spectroscopy (FTIR, Vertex 70, Bruker, Germany) in attenuated total reflectance (ATR) mode, within the spectral range of 4000–600 cm^−1^ and a resolution of 4 cm^−1^. In addition, XPS (Thermo ESCALAB 250Xi, USA) was utilized to determine the surface chemical states and elemental valence information of the scaffold surfaces.

### 2.3 In vitro degradation and electrochemical tests

*In vitro* degradation behavior of the scaffolds was evaluated in centrifuge tubes containing Hank's SBF at 37 °C over a period of 28 days. The volume of SBF was adjusted to 20 mL per 1 cm^2^ of scaffold surface area. Surface morphology after degradation was examined using SEM. To monitor the degradation process, pH values of the immersion medium were recorded daily during the first 14 days and every two days thereafter. At designated time points (1, 3, 7, 14, and 28 days), corrosion products were removed using a CrO_3_ solution (15 wt% in deionized water, 5 min under ultrasound conditions), and the mass loss was measured. Additionally, the residual scaffolds in the immersion solutions were digested in HNO_3_, and the concentrations of released metal ions were quantified using inductively coupled plasma optical emission spectrometry (ICP-OES, Agilent 5110, USA).

Mechanical performance degradation during immersion was assessed via compression test using a universal testing machine (Instron, equipped with a 10 kN load cell). Tests were conducted at room temperature, with a crosshead speed of 1 mm/min and a fixed compression height of 2.5 mm. Three groups of samples were tested following immersion durations of 0, 1, 3, 7, and 14 days.

To evaluate the instantaneous corrosion behavior, electrochemical tests were performed using a three-electrode system electrochemical workstation in Hank's SBF at 37 °C. Samples were first immersed for 60 min to stabilize the open-circuit potential (OCP), after which electrochemical impedance spectroscopy (EIS) was conducted across a frequency range of 10^−2^ to 10^5^ Hz with a 10 mV amplitude. Potentiodynamic polarization (PDP) tests were subsequently carried out to determine the corrosion potential (E_corr_) and corrosion current density (I_corr_), with values calculated using the Tafel extrapolation method.

### 2.4 Preparation and characterization of NRP-1-loaded liposomes

Liposomes were prepared using a conventional thin-film hydration method. Briefly, 1,2-dipalmitoyl-sn-glycero-3-phosphocholine (DPPC; Avanti Polar Lipids, USA) and cholesterol (Sigma-Aldrich, USA) were dissolved in chloroform at a molar ratio of 4:1. The solvent was removed under reduced pressure using a rotary evaporator (Heidolph, Germany) to form a uniform lipid film. The dried film was hydrated with deionized water at 55 °C for 30 min to obtain multilamellar vesicles. The suspension was then sequentially extruded through polycarbonate membranes with pore sizes of 200 nm and 100 nm using a mini-extruder (Avanti Polar Lipids, USA) to reduce particle size and improve uniformity.

For the preparation of NRP-1-loaded liposomes, recombinant human NRP-1 protein (Sino Biological, China) was added to the hydration medium at final concentrations of 5, 10, or 20 nM prior to extrusion. The suspensions were subsequently vortexed and sonicated (Branson, USA) to facilitate encapsulation and ensure homogeneity.

Particle size and polydispersity index (PDI) were determined by dynamic light scattering (DLS) using a Zetasizer Nano ZS90 analyzer (Malvern Instruments, UK) at 25 °C after 10-fold dilution with deionized water. Each sample was measured in triplicate. Liposome morphology was observed by scanning electron microscopy (SEM; Hitachi SU8010, Japan). Samples were deposited onto silicon wafers, air-dried, sputter-coated with gold, and imaged at an accelerating voltage of 10 kV.

### 2.5 Evaluation of encapsulation efficiency and release behavior

To determine the encapsulation efficiency (EE) of NRP-1-loaded liposomes, three liposomal formulations were prepared with initial NRP-1 concentrations of 5 nM, 10 nM, and 20 nM, respectively. After liposome formation, the suspensions were allowed to equilibrate to ensure complete protein–lipid association. Free (non-encapsulated) NRP-1 was then separated from the liposome-associated fraction using Amicon Ultra-4 centrifugal filter units (MWCO 10 kDa; Millipore, USA), which permit the passage of unencapsulated NRP-1 while retaining intact liposomes. Centrifugation was performed at 5000×*g* for 30 min at 4 °C to minimize potential protein degradation and liposome disruption. The filtrate containing free NRP-1 was carefully collected, and the concentration of unencapsulated NRP-1 was quantified using a human NRP-1 ELISA kit (Elabscience, China) following the manufacturer's protocol. Calibration curves were generated using known concentrations of NRP-1 to ensure accurate quantification. All measurements were conducted in triplicate to ensure reproducibility.

To evaluate the release kinetics, 1 mL of NRP-1-loaded lipos (5, 10, or 20 nM NRP-1) was loaded into dialysis bags (MWCO 10 kDa; Solarbio, China) and immersed in 20 mL PBS (pH 7.4) at 37 °C under gentle shaking (100 rpm). At daily intervals from day 0 to day 10, 1 mL of the medium was collected and replaced with fresh PBS. Each collected sample was dried at 57 °C in a metal bath, redissolved in 1 mL PBS and 0.5 mL methanol, and filtered through 0.22 μm membranes (Millipore, USA) before HPLC analysis. NRP-1 quantification was performed on a C18 column (4.6 × 250 mm) using a diode array detector (DAD) at 275 nm. The mobile phase comprised acetonitrile and 0.1% phosphoric acid (C:D = 60:40), ramped to 95:5 in 8 min, held for 2 min, and returned to baseline. The flow rate was 1.0 mL/min, column temperature 30 °C, and injection volume 10 μL. Concentrations were determined using a standard calibration curve.

### 2.6 Surface interaction and structural characterization

The surface charge of Lipo and NRP-1-loaded liposomes was measured using a Zetasizer Nano ZS90 analyzer (Malvern Instruments, UK) at 25 °C after 10-fold dilution with deionized water. Each sample was measured three times and the average value was recorded. To evaluate liposome–coating interactions, LDH- and PDA-coated substrates were incubated with NRP-1-loaded liposome suspensions. After incubation, samples were gently rinsed with deionized water to remove unbound liposomes and dried under nitrogen flow. The zeta potential of the coated substrates was then measured using an electrokinetic analyzer (SurPASS 3, Anton Paar, Austria), with measurements taken at three different positions to ensure reproducibility.

Fourier transform infrared (FTIR) spectroscopy was performed using a Nicolet iS50 spectrometer (Thermo Fisher Scientific, USA) in the range of 4000–500 cm^−1^ with a resolution of 4 cm^−1^ to analyze the chemical characteristics of liposome-coated samples (HTO-LIPO, HTO-LDH-LIPO, and HTO-LDH-PDA-LIPO). Samples were vacuum-dried prior to measurement and spectra were averaged over 32 scans. In addition, Lipo and NRP-1-loaded liposomes were labeled with FITC and incubated with the samples for 2 h at room temperature. After PBS washing, fluorescence distribution was observed using confocal laser scanning microscopy (Leica TCS SP8, Germany; excitation 488 nm). Fluorescence co-localization was quantified using the Coloc 2 plugin in ImageJ to evaluate liposome adsorption and distribution on the coatings.

### 2.7 Cell culture

BMSCs were isolated from 2- to 3-month-old specific-pathogen-free (SPF) New Zealand white rabbits. Bilateral tibiae and femora were harvested under sterile conditions, and BMSCs were obtained using the whole bone marrow adherence method. Cells were cultured in Low-Glucose Dulbecco's Modified Eagle Medium (L-DMEM; Gibco, USA) supplemented with 10% (v/v) fetal bovine serum (FBS; Gibco, USA) and 1% (v/v) penicillin–streptomycin (Gibco, USA). Cultures were maintained at 37 °C in a humidified atmosphere containing 5% CO_2_ (Thermo Scientific, USA). When cell confluence reached approximately 80%, subculture was performed using EDTA solution (Gibco, USA) for enzymatic detachment. The medium was refreshed every two days. BMSCs at passage 3 were used for all subsequent experiments.

The murine macrophage cell line RAW264.7 (ATCC, USA) was cultured in DMEM supplemented with 10% FBS and 1% penicillin–streptomycin at 37 °C in a humidified atmosphere containing 5% CO_2_. The culture medium was replaced every 2–3 days.

HUVECs (ScienCell Research Laboratories, USA) were cultured in endothelial cell growth medium supplemented with endothelial growth factors in accordance with the manufacturer's instructions. Cells were maintained at 37 °C in a humidified incubator with 5% CO_2_, and the culture medium was changed every 2 days. HUVECs at passages 3–6 were used for all experiments.

### 2.8 Preparation of Mg^2+^ extracts and cytocompatibility evaluation

Porous Gyroid-structured Mg alloy scaffolds (diameter 5 mm, height 5 mm; surface area ≈277 mm^2^) were used to prepare Mg^2+^ extracts according to the international standard GB/T 16886.12-2017/ISO 10993-12:2012 at a surface area–to–volume ratio of 1.25 cm^2^ ml^−1^. Scaffolds were incubated in serum-free medium at 37 °C with 5% CO_2_ for 72 h. After incubation, the Mg alloy samples were removed and the extracts were filtered through a 0.22 μm membrane to eliminate debris. Finally, 10% (w/v) FBS and 1% (w/v) antibiotics were added to obtain the Mg^2+^ extract medium.

For cytocompatibility evaluation, all scaffolds were sterilized by immersion in 75% ethanol for 2 h followed by UV irradiation for 30 min on each side. BMSCs were seeded onto the scaffolds at a density of 1 × 10^5^ cells per scaffold and cultured in α-MEM supplemented with 10% FBS and 1% penicillin–streptomycin (Gibco, USA) at 37 °C under 5% CO_2_ in 24-well ultra-low attachment plates (Corning, USA). After 7 days, cell adhesion and distribution were examined by confocal laser scanning microscopy after cytoskeletal staining (nuclei: blue; cytoskeleton: green). Images were acquired using a Nikon A1R confocal microscope and analyzed with ImageJ to quantify cell number per 5 × 10^5^ μm^2^ and average cell area from three random regions.

Cell viability was assessed using a CCK-8 assay (Dojindo, Japan). Briefly, 10% CCK-8 reagent was added after 7 days of culture and incubated for 2 h, followed by absorbance measurement at 450 nm using a microplate reader (BioTek, USA). Cell morphology on the scaffolds was further observed by SEM after fixation, graded ethanol dehydration (30–100%), air drying, and gold sputter-coating. Imaging was performed using a Zeiss GeminiSEM 300 (Carl Zeiss, Germany) at an accelerating voltage of 5 kV.

### 2.9 In vitro immunomodulatory evaluation and flow cytometric analysis

RAW264.7 murine macrophages (ATCC® TIB-71™, USA) were used to evaluate the anti-inflammatory effects of Mg^2+^. Cells were cultured in DMEM supplemented with 10% FBS and 1% penicillin–streptomycin (Gibco, USA) at 37 °C with 5% CO_2_. Five experimental groups were established: NC group (untreated control), M1 group stimulated with lipopolysaccharide (LPS, 100 ng mL^−1^; Sigma-Aldrich, USA) and interferon-γ (IFN-γ, 20 ng mL^−1^; PeproTech, USA) for 24 h, M2 group treated with interleukin-4 (IL-4, 20 ng mL^−1^; PeproTech, USA) for 24 h, Mg group cultured in Mg^2+^ extracts, and M1+Mg group in which cells were first stimulated with LPS and IFN-γ for 12 h followed by incubation with Mg^2+^ extracts for 24 h.

Macrophage polarization was analyzed by flow cytometry at 24, 48, and 72 h. Cells were harvested, blocked with anti-CD16/32 (BioLegend) at 4 °C for 15 min, and stained with LIVE/DEAD Fixable Viability Dye (Thermo Fisher Scientific) to exclude nonviable cells. Surface markers were labeled using anti-CD86-FITC (M1 marker) and anti-CD206-PE (M2 marker) antibodies (BioLegend, 1:100) at 4 °C for 30 min in the dark. After washing, cells were resuspended in PBS and analyzed using a CytoFLEX flow cytometer (Beckman Coulter), collecting at least 10,000 events per sample. Data were processed using FlowJo software (BD Biosciences, V10.8.1), with unstained, single-stained, and fluorescence minus one (FMO) controls used for gating.

In addition, flow cytometry was performed to evaluate potential phenotypic changes in BMSCs after Mg^2+^ exposure. BMSCs cultured under standard conditions or in Mg^2+^-enriched medium for 24 h were harvested, washed with PBS, and incubated with fluorochrome-conjugated antibodies against CD34, CD44, CD90, and CD29 (Bioscience, USA) at 4 °C for 30 min in the dark. After washing, cells were analyzed by flow cytometry, and surface marker expression profiles were compared between Mg^2+^-treated and untreated groups.

### 2.10 Gene and protein expression analysis

Protein and mRNA expression levels were analyzed by western blotting and quantitative real-time PCR (qRT-PCR). For gene silencing experiments, small interfering RNAs targeting ID1 (si-ID1) and NR4A1 (si-NR4A1), together with a negative control siRNA (si-NC), were obtained from GenePharma (Shanghai, China) and transfected into cells using Lipofectamine™ 3000 (Invitrogen, USA). After 48 h, cells were collected for subsequent analysis.

For western blotting, total protein was extracted using RIPA lysis buffer containing protease inhibitors. Equal amounts of protein were separated by SDS-PAGE, transferred onto PVDF membranes, and incubated with primary antibodies against iNOS, TNF-α, Arg1, IL-10, BMP-2, COL-I, ID1, VEGFA, FGF2, and NR4A1. After incubation with HRP-conjugated secondary antibodies, protein bands were visualized using an enhanced chemiluminescence system and quantified with ImageJ. Protein expression levels were normalized to GAPDH or β-actin.

For qRT-PCR analysis, total RNA was extracted using TRIzol reagent and reverse-transcribed into cDNA. Quantitative PCR was performed using a real-time PCR system (LightCycler480), and relative gene expression was calculated using the 2^−ΔΔCt method with GAPDH as the internal reference. Primer sequences are listed in Table S3–S5. Knockdown efficiency of ID1 and NR4A1 was confirmed by qRT-PCR after 48 h of siRNA transfection.

### 2.11 Osteogenic differentiation and immunofluorescence analysis

BMSCs were cultured in osteogenic induction medium containing L-DMEM supplemented with 10% FBS, 1% penicillin–streptomycin, 10 mM β-glycerophosphate, 50 μg mL^−1^ ascorbic acid, and 100 nM dexamethasone. Three experimental groups were established: NC group (osteogenic medium only), Mg group (osteogenic medium supplemented with Mg^2+^ extract), and Mg + NRP-1 group (Mg group additionally supplemented with 10 nM NRP-1). Cells were seeded in 24-well plates at a density of 1 × 10^4^ cells cm^−2^ and cultured at 37 °C with 5% CO_2_, with medium refreshed every two days.

Osteogenic differentiation was evaluated by alkaline phosphatase (ALP) staining on day 14 and Alizarin Red S (ARS) staining on day 21 using commercial kits. Relative ALP activity was quantified, and mineralized matrix deposition was further assessed by dissolving ARS-stained calcium nodules with cetylpyridinium chloride followed by absorbance measurement at 562 nm.

In addition, immunofluorescence staining was performed to assess the expression of osteogenic and angiogenic proteins. BMP-2 and COL-I were examined on day 21, while VEGFA and FGF2 were analyzed on day 14. After fixation, cells were incubated with primary antibodies and fluorescent secondary antibodies, with nuclei counterstained by DAPI and cytoskeleton labeled by phalloidin. Fluorescence images were captured using confocal laser scanning microscopy, and ImageJ was used for semi-quantitative analysis of fluorescence intensity.

### 2.12 Cell migration and angiogenesis assays

BMSC migration was evaluated using scratch and Transwell assays. For the scratch assay, BMSCs were seeded in six-well plates (4 × 10^5^ cells per well). After reaching confluence, a linear wound was created using a 1 mL pipette tip under serum-free conditions. Images were captured at 0, 24, and 48 h using an inverted microscope (Zeiss, Germany), and the wound closure area was quantified using ImageJ.

For the Transwell assay, BMSCs were seeded in the upper chambers of Transwell inserts (8.0 μm pore size; Servicebio, China), while different culture conditions were applied in the lower chambers according to the experimental groups. After 24 and 48 h, migrated cells on the lower membrane surface were fixed, stained with crystal violet, and quantified using ImageJ.

Angiogenic potential was further assessed by a Matrigel tube formation assay. Growth factor–reduced Matrigel (Corning, USA) was polymerized in 96-well plates, and HUVECs were used as responder cells. Four groups were established: NC (HUVECs alone), Co (HUVECs co-cultured with untreated BMSCs), Mg + Co (HUVECs co-cultured with BMSCs pretreated with Mg^2+^ extract), and Mg + Co + VEGF + inhibit (co-culture with VEGF inhibitor). BMSCs were seeded in Transwell inserts to allow paracrine signaling without direct contact. For VEGF blockade, recombinant human VEGFR1/Flt-1 Fc chimera protein (R&D Systems) was added to the medium. After co-culture, HUVECs were seeded onto Matrigel and tube formation was observed at 12 and 24 h. Total tube length, junctions, and mesh numbers were quantified using the Angiogenesis Analyzer plugin in ImageJ.

### 2.13 Establishment of ischemic bone defect model in rabbits

Female New Zealand white rabbits (2.0–2.5 kg) were obtained from Zhongjian Huatongwei Animal Research Center. All procedures were approved by the Institutional Animal Care and Use Committee (Ethical Approval No. QRB219-21). Under general inhalation anesthesia, the medial proximal femoral region was shaved and disinfected 24 h before surgery. A 2–3 cm longitudinal incision was made to expose the femoral artery, and approximately 1.5 cm of the vessel was bluntly isolated and ligated proximally and distally to block blood flow (Fig. S7). After 15 min, laser speckle contrast imaging (LSCI) was performed on the lateral femoral condyle to confirm reduced perfusion in the ischemic limb.

Subsequently, a cylindrical bone defect (5 mm diameter × 5 mm depth) was created in the lateral femoral condyle using a surgical drill with a depth limiter. Different scaffolds were implanted according to the experimental groups, and the incision was closed layer by layer. Postoperatively, rabbits received intramuscular penicillin sodium (40,000 IU kg^−1^ day^−1^) for three days to prevent infection.

### 2.14 Imaging analysis

LSCI was used to evaluate tissue perfusion in the lateral femoral condyle following femoral artery ligation. Under isoflurane anesthesia, the lateral knee region was surgically exposed and imaged using a laser speckle perfusion system (PeriCam PSI System, Perimed AB, Sweden; 785 nm). Perfusion images were analyzed with PeriSoft software to quantify regional blood flow and confirm the ischemic status of the hindlimb.

. Bone regeneration was assessed at 6 and 12 weeks post-implantation using X-ray imaging (Bruker FX Pro, Bruker, Germany) and micro-computed tomography (Micro-CT; Skyscan 1176, Bruker, Belgium) with a resolution of 35 μm. A cylindrical region of interest (5.5 mm in diameter and depth) centered on the defect site was selected for analysis. Three-dimensional reconstruction was performed to visualize the scaffold and newly formed bone. Trabecular bone parameters, including bone volume fraction (BV/TV), trabecular number (Tb.N), trabecular thickness (Tb.Th), and trabecular separation (Tb.Sp), were quantitatively calculated.

### 2.15 Histological staining and immunofluorescence analysis

At postoperative 6 and 12 weeks, rabbits were euthanized, and the distal femoral condyles from the operated side were harvested intact. Samples designated for hard tissue sectioning were fixed, dehydrated, and embedded in resin, followed by preparation of ground sections (approximately 100 μm thick) using a hard tissue slicing system. Standard hematoxylin and eosin (HE) staining and Masson's trichrome staining were then performed to evaluate tissue morphology, collagen fiber distribution, and new bone formation.

To further assess neovascularization within the defect area, additional femoral condyle specimens were decalcified, paraffin-embedded, and sectioned at 5 μm thickness. Immunofluorescence staining was conducted to detect vascularization-related markers, including CD31 and VEGF. Primary antibodies included anti-CD31 (ab28364, Abcam, UK) and anti-VEGF (ab32152, Abcam, UK), both diluted at 1:200. DAPI was used for nuclear counterstaining. Fluorescence images were captured using a laser scanning confocal microscope, and fluorescence intensity was semi-quantitatively analyzed using ImageJ software.

### 2.16 RNA sequencing and data analysis

Three groups of cell samples were prepared for treatment. The first group (NC) involved BMSCs cultured under standard adherent conditions for 3 days at a density of 5 × 10^5^ cells/well. In the second group (Mg), BMSCs (5 × 10^5^ cells/well) were cultured for 21 days in medium containing Mg^2+^ extract (1.25 cm^2^/mL). The third group (Mg + NRP-1) involved BMSCs (5 × 10^5^ cells/well) cultured in Mg^2+^ extract-containing medium supplemented with 10 nM NRP-1 for 21 days. Each group was conducted in triplicate. Total RNA was extracted using Trizol reagent (Servicebio, China), and transcriptome sequencing was performed on the BGISEQ-500 platform. Differentially expressed genes (DEGs) were identified using the DESeq2 algorithm, with P-values adjusted using the False Discovery Rate (FDR) method. Gene enrichment analyses, including GO, KEGG, and GSEA, were performed to explore the biological significance of DEGs. A threshold of p < 0.05 was considered statistically significant for all enrichment analyses. The Principal Component Analysis (PCA), RNA-Seq correlation test, and Cluster analysis of differentially expressed genes can be found in Fig. S8, S9 and S10.

### 2.17 Immunohistochemical validation of osteogenic and angiogenic pathways *in vivo*

A rabbit lower-limb ischemic bone defect model was established and divided into three groups: (1) Control group, defects filled with bone wax without implants; (2) Mg group, defects implanted with unmodified Mg scaffolds; and (3) Mg–NRP-1 group, defects implanted with NRP-1-modified Mg scaffolds. Animals were sacrificed on postoperative day 21, and tissues from the defect region were harvested, fixed, paraffin-embedded, and sectioned (5 μm) for immunohistochemical staining.

To evaluate osteogenic and angiogenic signaling pathways, BMPR1 and p-SMAD1/5/8 were analyzed as markers of the TGF-β/SMAD pathway, while p-PI3K and p-AKT were used to assess the PI3K/AKT pathway. Sections were incubated with the corresponding primary antibodies (1:200), followed by HRP-conjugated secondary antibodies. Immunoreactivity was visualized using DAB chromogen, with positive staining appearing brownish-yellow.

### 2.18 Statistical analysis

All quantitative data are presented as mean ± standard deviation (SD) with a sample size of n ≧ 3. Statistical analysis was performed using one-way analysis of variance (ANOVA) followed by Tukey's test. Differences were considered statistically significant at ∗p < 0.05, highly significant at ∗∗p < 0.01, and extremely significant at ∗∗∗p < 0.001.

## 3 Results and discussion

### 3.1 Formation and characterization of surface coatings on Mg alloys

**Fig. 2:**
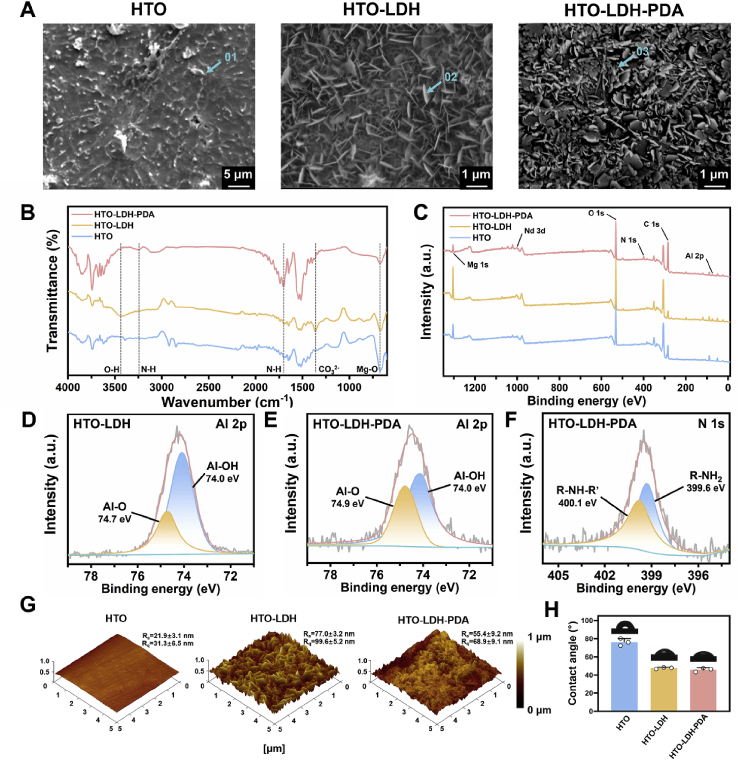
Synthesis and characterization of the composite coatings. (**A**) Surface morphology, (**B**) ATR-FTIR and (**C**) XPS spectra of the three layers. (**D**) Al 2p spectrum of HTO-LDH. (**E**) Al 2p spectrum of HTO-LDH-PDA. (**F**) N 1s spectrum of HTO-LDH-PDA. (**G**) AFM images and (**H**) Water contact angles of the three layers. Data are presented as mean values ± s.d. (n = 3).

The synthesis of Mg alloy scaffold with composite coatings was carried out in three sequential steps. First, the scaffolds were printed by LPBF, polished in acid to remove the attached powder particles, resulting in the as-printed scaffolds. These scaffolds were subsequently exposed in flowing air at 525 °C for 8 h to obtain HTO layer. As shown in the coating morphology in Fig. 2A, Fig. S4 and energy-dispersive X-ray spectroscopy (EDX) in Table S1, the concentrations of Y, Nd, and O on the HTO scaffold surface were significantly higher than those in the as-printed scaffold surface. The main components were identified as Y_2_O_3_, Nd_2_O_3_, and MgO, indicating the formation of an oxide film approximately 1.5–2 μm thick during the oxidation process. Next, LDH were formed on the surface via hydrothermal treatment at 120 °C for 6 h, exhibiting nanoscale sheet structures and a trace amount of Al detected by EDX on the surface. For more information regarding the characterization of HTO and LDH layers, please refer to our previous work [24,38,39]. Finally, a PDA layer was deposited on the LDH surface by co-stirring with dopamine solution for 3 h. Microscopically, the addition of PDA altered the nanosheet morphology into flat, petal-like structures. The formation of hydrogen bonds between PDA and LDH contributed to the uniformity and stability of the coating.

Fig. 2B presents the Attenuated Total Reflectance – Fourier Transform Infrared (ATR-FTIR) spectrum of the samples. In the HTO–LDH surface, characteristic stretching vibrations were observed at 1361 cm^−1^ and 3433 cm^−1^, corresponding to CO_3_^2−^ and O–H groups respectively, confirming the successful formation of Mg–Al–CO_3_ LDH. In contrast, the HTO–LDH–PDA surface exhibited distinct absorption bands at 1703 cm^−1^ and 3246 cm^−1^, corresponding to N–H bonds, indicating successful polymerization of PDA. The signals associated with the LDH structure in the HTO–LDH–PDA surface were relatively weak, indicating the coverage of PDA at the top of LDH layer. Fig. S5 presents the X-ray diffraction (XRD) patterns of the samples. Distinct signals corresponding to the characteristic peaks of Y_2_O_3_ and Nd_2_O_3_ from the HTO layer, as well as the (003) and (006) crystal planes of the LDH phase, are clearly visible in the respective samples. It should be noted that, due to the amorphous nature of PDA, the XRD pattern of the HTO-LDH-PDA sample closely resembles that of the HTO-LDH sample. As shown in Fig. 2C–F, X-ray photoelectron spectroscopy (XPS) analysis further verified the presence of Al-OH on both the HTO–LDH and HTO–LDH–PDA surfaces (binding energy at ∼74.0 eV), and the presence of R-NH-R’ on the HTO–LDH–PDA surface (binding energy at ∼400.1 eV), confirming the successful stepwise formation and stable layering of the composite coatings.

As shown in Fig. 2G, tapping mode atomic force microscopy (AFM) revealed that the plate-like LDH structure markedly increased the surface roughness, with the average roughness (Ra) rising from 21.9 ± 3.1 nm to 77.0 ± 3.2 nm. The subsequent introduction of PDA slightly reduced the roughness. Compared to the HTO surface, the increased roughness of the HTO–LDH surface led to enhanced hydrophilicity, as evidenced by a decrease in the water contact angle (CA) from 76.2 ± 3.2° to 47.7 ± 0.8°. In the HTO–LDH–PDA group, the PDA layer further contributed to surface wettability due to the presence of hydrophilic functional groups (–OH, –NH_2_), resulting in a CA of 45.8 ± 1.8° (Fig. 2H). These surface improvements are regarded beneficial for cell adhesion, proliferation, and integration.

### 3.2 Improved in vitro degradation behavior regulated by composite coatings

The degradation behavior of the Mg alloy scaffold was evaluated by continuous immersion in Hank's simulated body fluid (SBF) at 37 °C for 28 days. Fig. S1 shows the degradation levels of the three scaffold groups after HTO, HTO–LDH and HTO–LDH–PDA, respectively. Following the application of the LDH layer, the degradation rate was significantly reduced, and the scaffold maintained its structural integrity for approximately 14 days. The degradation behavior of the HTO-LDH-PDA group was comparable to that of the HTO-LDH group.

**Fig. 3:**
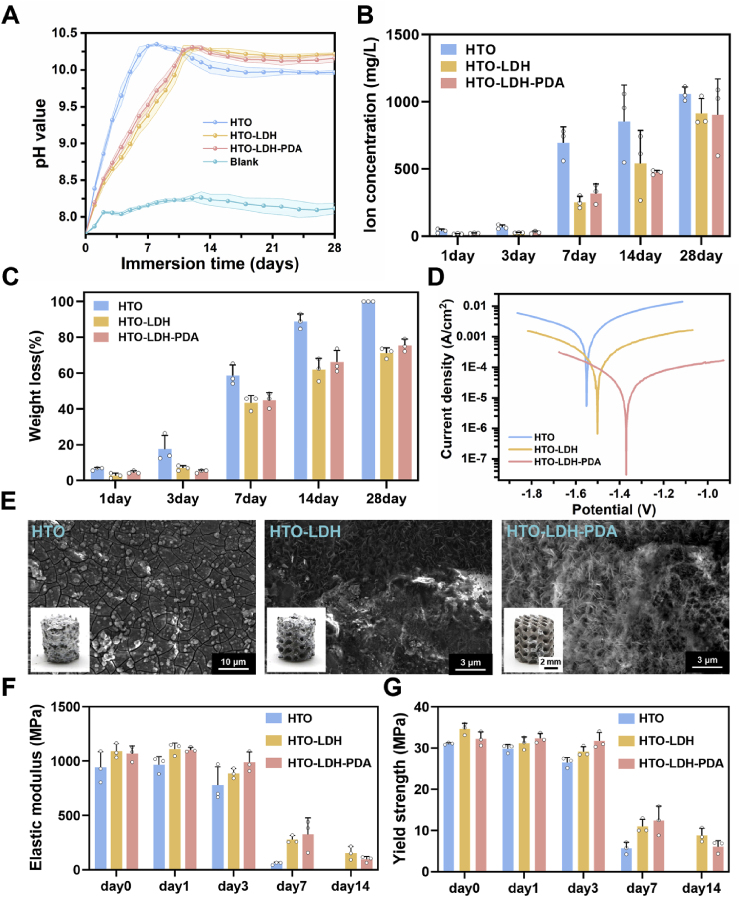
Degradation behavior of Mg scaffolds after different coating processes. (**A**) pH values of immersion solutions. (**B**) Mg^2+^ concentrations after 1, 3, 7, 14, and 28 days of immersion. (**C**) Scaffold weight loss rates. (**D**) PDP curves. (**E**) Microstructural morphologies of the scaffold surfaces after 7 days of immersion. (**F**) Compressive elastic modulus. (**G**) Compressive yield strength of the scaffolds after 0, 1, 3, 7, and 14 days of immersion. All measurement data are presented as mean values ± s.d. (n = 3).

Further monitoring of pH variation, Mg^2+^ ion release, and weight loss rate during the immersion period was conducted (Fig. 3A–C). The HTO group reached a peak pH of 10.32 ± 0.02 at 7 days. Correspondingly, Mg^2+^ concentration and weight loss also showed significant stepwise increases from day 3 to day 7 (rising from 66.4 ± 13.7 mg/L to 695.0 ± 97.2 mg/L and from 17.59 ± 6.24% to 58.80 ± 4.72%, respectively). In contrast, the HTO-LDH and HTO-LDH-PDA groups exhibited more stable degradation. Their pH values peaked around day 13, and Mg^2+^ release at 7 days reduced by 63.73% and 54.43% respectively compared to the HTO group. The corresponding weight loss rates at 7 days were 43.52 ± 3.27% and 44.91 ± 3.46%, and the values gradually increased to 71.30 ± 2.36% and 75.46 ± 2.85% over the subsequent 21 days, demonstrating a pronounced degradation-retarding effect. Notably, the amount of ion release in this research, including potentially biologically toxic Al, was insufficient to cause changes in the osmotic pressure of the physiological environment or raise biosafety concerns. Electrochemical testing provides a direct assessment of the passivation properties of surface coatings. Fig. 3D shows the Potentiodynamic polarization (PDP) curves of the three scaffold groups. With the coatings were attached layer by layer, both the self-corrosion potential (E_corr_) and self-corrosion current (I_corr_) shifted in directions of enhanced corrosion protection. The E_corr_ value of the HTO, HTO-LDH, and HTO-LDH-PDA groups were −1.54 ± 0.02 V, −1.47 ± 0.03 V, and −1.37 ± 0.05 V, respectively, while the corresponding corrosion currents were 3.35 ± 1.50 × 10^−4^ A/cm^2^, 4.74 ± 2.09 × 10^−5^ A/cm^2^, and 2.02 ± 1.37 × 10^−5^ A/cm^2^. Fig. S3 and Table S2 present the Electrochemical Impedance Spectroscopy (EIS) results and the corresponding equivalent circuit fitting data, respectively. Both low-frequency impedance in Bode plot and R_1_ value in Nyquist plot of the samples increased progressively with the deposition of each coating layer. A particularly significant enhancement was observed after the introduction of the LDH layer, further verifying that the multilayer coating effectively strengthens the passivation of the magnesium alloy.

As shown in Fig. 3E, scanning electron microscopy (SEM) observations of the scaffold surface after 7 days of in vitro immersion revealed distinct morphological differences among the groups. The surface of the HTO scaffold was extensively covered with corrosion products, including Mg(OH)_2_ and blocky Ca–P precipitates, indicating a significantly more severe and accelerated corrosion behavior compared with the other groups. In contrast, only a small amount of corrosion products was deposited on the surfaces of both the HTO–LDH and HTO–LDH–PDA scaffolds, where the characteristic nano-sheet structures remained clearly visible, demonstrating the sustained protective effect of the coating at this stage. Furthermore, the compressive strength and Young's modulus were measured to evaluate its load-bearing capacity during degradation. As shown in Fig. 3F and G and Fig. S6, the mechanical properties of the samples coated with HTO-LDH and HTO-LDH-PDA layers exhibited a slight improvement compared to the HTO samples. This enhancement is attributed to the hydrothermal treatment, which acts similarly to a low-temperature aging process and possibly contributes to microstructural strengthening. During degradation, the yield strength and Young's modulus of the HTO group decreased rapidly to 5.69 ± 1.18 MPa and 57.78 ± 10.28 MPa respectively by day 7. In contrast, the HTO-LDH and HTO-LDH-PDA groups retained significantly higher mechanical performance over the same period and only reached similarly low levels by day 14. This direct correlation between the slower degradation and the prolonged retention of mechanical properties underscores the critical role of the multilayer coating in sustaining structural stability.

The oxide and LDH layers formed through the HTO process and subsequent hydrothermal treatment have demonstrated a positive effect on inhibiting the corrosion of Mg alloys [38]: The protective layers formed by the HTO and LDH processes are highly dense, effectively isolating the Mg matrix from body fluids and thereby reducing the rate of galvanic corrosion. Additionally, the hydrophilic nanosheet morphology by LDH contributes to promoted surface roughness, thereby enhancing cell adhesion. The CO_3_^2−^ anions intercalated within the LDH layer exert a repulsive force against penetration of Cl^−^ into the matrix, further mitigating corrosion. The PDA coating grows stably on the LDH layer through strong hydrogen-bonding interactions, ensuring uniform and adherent coverage. In addition, it provides a modest supplementary passivation effect that further restrains the initial degradation of the substrate. More importantly, the intrinsically hydrophilic and bioactive PDA interface establishes a favorable microenvironment for cell adhesion and extracellular matrix deposition, thereby laying a critical foundation for the subsequent outstanding biological performance of the magnesium scaffold.

It is worth mentioning that the formation of the three-layer coating is also interdependent. For comparison, a magnesium-based LDH coating without the HTO layer and an HTO–PDA coating without the LDH layer were prepared separately, as shown in Fig. S7. In the absence of the HTO layer, the magnesium-based LDH coating suffered from premature corrosion during the hydrothermal process, as the magnesium substrate lacked the basic protection provided by HTO. Conversely, the HTO–PDA coating without the LDH layer was prone to the formation of surface defects due to the weak interfacial bonding. These results demonstrate that the three-layer coating system is interconnected, with each layer playing an indispensable role in ensuring coating integrity and stability.

### 3.3 Characterization of Lipo and NRP-1-loaded lipos

**Fig. 4:**
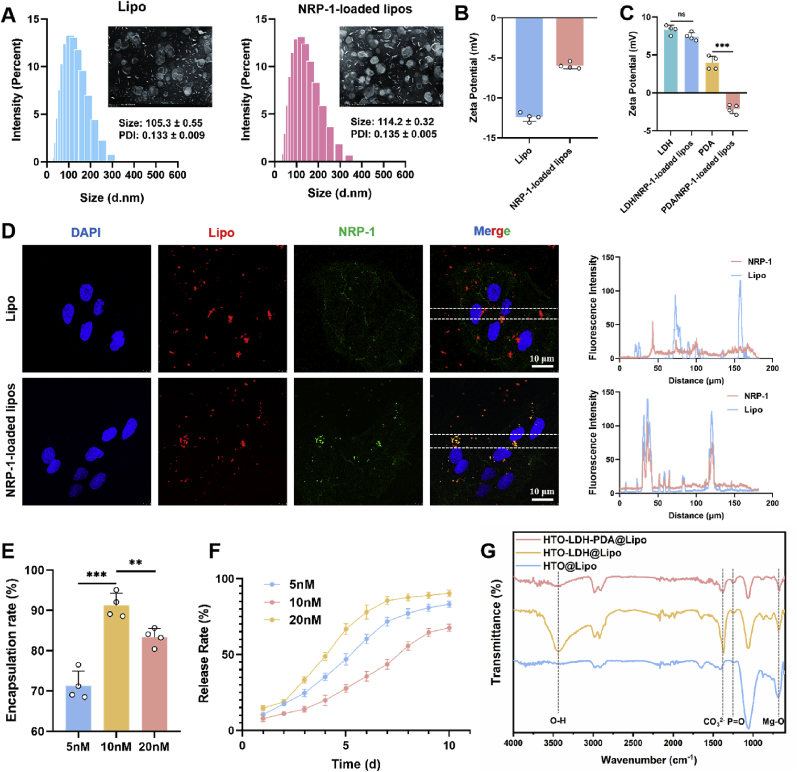
Characterization of Lipo and NRP-1-loaded lipos. (**A**) Characterization of Lipo and NRP-1-loaded lipos by SEM, Particle Size, and PDI. (**B-C**) Quantitative analysis of Zeta Potential in different groups. Data are presented as mean values ± s.d. (n = 4). (**D**) Immunofluorescence imaging and quantitative co-localization analysis of Lipo and NRP-1-loaded lipos. (**E-F**) Quantitative analysis of encapsulation efficiency and release profiles of NRP-1 at three different concentrations. Data are presented as mean values ± s.d. (n = 4). (**G**) FTIR characterization of coatings from three experimental groups. ns, no significant (p > 0.05), ∗p < 0.05, ∗∗p < 0.01, ∗∗∗p < 0.001 (one-way ANOVA).

SEM observation revealed that both Lipo and NRP-1-loaded lipos exhibited uniform, spherical-like morphologies with smooth surfaces. The average particle size of Lipo was 105.3 ± 0.55 nm with a polydispersity index (PDI) of 0.133 ± 0.009. After encapsulation of NRP-1, the particle size of NRP-1-loaded lipos slightly increased to 114.2 ± 0.32 nm, while the PDI remained stable at 0.135 ± 0.005 (Fig. 4A), indicating high morphological stability and size uniformity. In addition, as shown in Fig. 4D, NRP-1-loaded lipos exhibited strong co-localization with FITC-labeled NRP-1, with a significantly higher overlap in fluorescence intensity profiles compared to the Lipo group. The narrow particle size distribution and high fluorescence co-localization demonstrate the colloidal stability and reliable NRP-1 loading efficiency of NRP-1-loaded lipos.

To optimize the encapsulation efficiency of NRP-1 in liposomes, three initial concentrations of NRP-1 (5 nM, 10 nM, and 20 nM) were tested. Among them, the 10 nM group achieving significantly efficiency than the other concentrations (Fig. 4E), suggesting that this concentration provided a more favorable condition for stable NRP-1 incorporation into liposomes. Furthermore, the release behavior of NRP-1 from NRP-1-loaded lipos at these three concentrations was evaluated in vitro. As shown in Fig. 4F, all groups exhibited a relatively rapid release within the first 7 days, followed by a gradual decrease in release rate. Notably, the 10 nM group reached a cumulative release of 92.2 ± 1.6% by day 10, demonstrating not only the highest release amount but also a smoother and more sustained release profile. This sustained release behavior is conducive to the prolonged bioavailability of NRP-1, thereby supporting its biological activity in tissue repair applications.

To evaluate the attachment efficiency of NRP-1-loaded lipos on different coatings, we first measured the zeta potential of Lipo and NRP-1-loaded lipos, which were −12.8 ± 1.6 mV and −5.4 ± 0.9 mV, respectively (Fig. 4B). The reduced negativity after NRP-1 loading suggests a slight alteration in the surface charge properties of the liposomes. Subsequently, NRP-1-loaded lipos was incubated with LDH and PDA-coated surfaces, and zeta potential measurements were performed before and after incubation to assess liposome adsorption. The LDH-coated group exhibited negligible changes in surface potential following NRP-1-loaded lipos exposure, indicating limited adsorption. In contrast, the surface potential of the PDA-coated group decreased significantly from +3.7 ± 1.4 mV to −2.7 ± 1.2 mV, suggesting substantial binding of negatively charged NRP-1-loaded lipos to the PDA coating (Fig. 4C). To further verify liposome adsorption, FT-IR spectroscopy was performed on the coated scaffolds after NRP-1-loaded lipos treatment (Fig. 4G). A marked increase in the intensity of the characteristic P=O stretching vibration (∼1230–1250 cm^−1^), corresponding to the phosphate ester groups in phospholipids, was observed in the HTO-LDH-PDA@Lipo group. This enhanced signal reflects a higher surface density of lipid molecules, indicating more effective liposome adhesion on the PDA-modified coating. Notably, the HTO-LDH@Lipo group exhibited stronger absorption peaks in the O–H and CO_3_^2−^ regions, which can be attributed to its hydrophilic surface and carbonate intercalation rather than liposome presence. In contrast, the Mg–O peak was most prominent in the HTO group and gradually diminished in LDH and PDA coatings, suggesting progressive coverage of the inorganic surface. Such surface masking, particularly with PDA, not only indicates efficient liposome adsorption but also contributes to the creation of a more biologically compatible interface, which is favorable for subsequent bioactive delivery and controlled release applications.

Collectively, these results demonstrate that the PDA coating exhibits superior adsorption capacity for NRP-1-loaded lipos compared to the conventional LDH coating. The abundant catechol and amine functional groups in PDA not only facilitate the formation of hydrogen bonds with the polar phosphate headgroups of liposomes, but also enhance electrostatic interactions, thereby promoting higher-density and more stable liposome attachment [40]. Additionally, the excellent surface wettability and interfacial activity of PDA contribute to the formation of a uniform nano-delivery interface [41]. It is worth noting that NRP-1, as a potent angiogenic modulator, requires stable surface immobilization to ensure its sustained release and effective targeted delivery [42]. The high surface reactivity and lipophilic moieties provided by PDA offer a favorable environment for the secure binding of NRP-1 while preserving its bioactivity, thus laying a solid foundation for the construction of functionalized delivery platforms.

### 3.4 Cytocompatibility evaluation of BMSCs cultured on different coatings

**Fig. 5:**
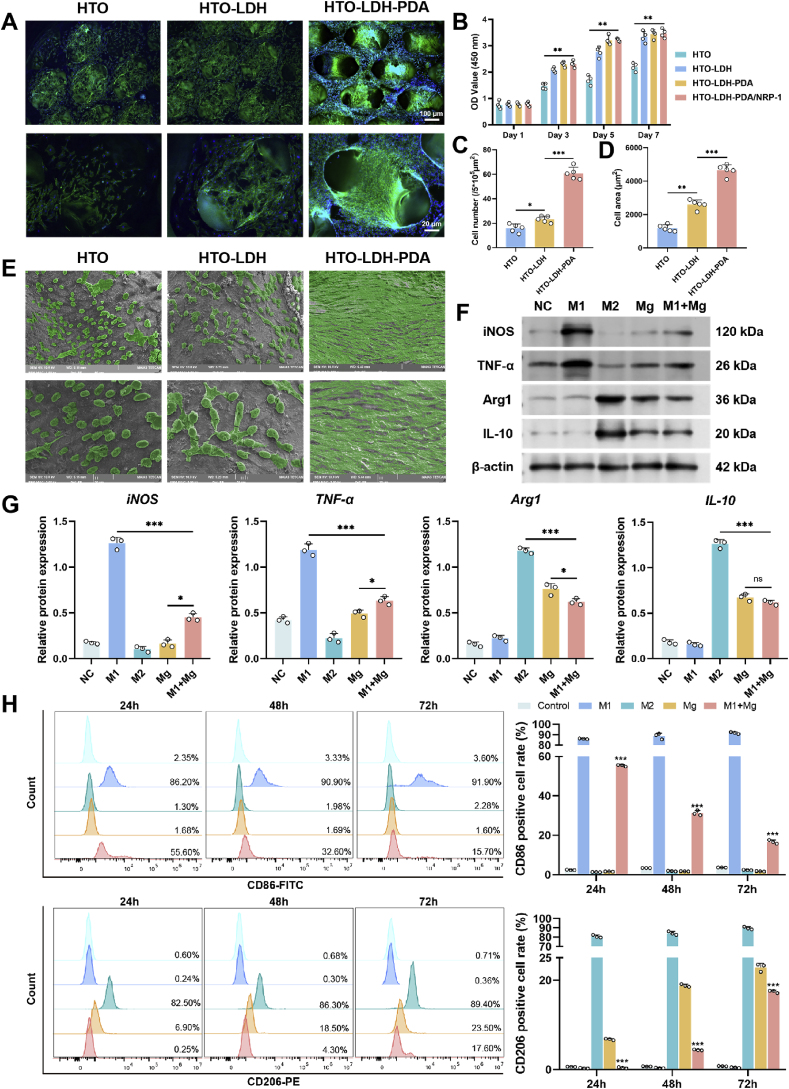
Cytocompatibility of coatings and immunoregulatory effects of Mg^2+^. (**A**) Three-Dimensional cytoskeletal imaging of BMSCs cultured on scaffolds with different coatings at day 7. (**B**) Cell viability of different groups assessed by CCK-8 assay on days 1, 3, 5, and 7. Data are presented as mean values ± s.d. (n = 4). (**C-D**) Quantitative analysis of cell number and spreading area. Data are presented as mean values ± s.d. (n = 5). (**E**) SEM images of BMSCs cultured for 3 days on three different coatings. (**F**) Western-blot analysis of iNOS, TNF-α, Arg1, and IL-10. (**G**) Quantitative analysis of relative protein expression of iNOS, TNF-α, Arg1, and IL-10. (**H**) Flow cytometry analysis of macrophage polarization at 24 h, 48 h, and 72 h. Data are presented as mean values ± s.d. (n = 3). ns, no significant (p > 0.05), ∗p < 0.05, ∗∗p < 0.01, ∗∗∗p < 0.001 (one-way ANOVA).

To assess the cytocompatibility of different coatings, Bone Marrow–Derived Mesenchymal Stem Cells (BMSCs) were cultured for 7 days on Diamond sheet Mg scaffolds modified with HTO, HTO-LDH, and HTO-LDH-PDA coatings. As shown in Fig. 5A, BMSCs adhered to the scaffold surfaces in all groups, however, the HTO-LDH-PDA group exhibited notably higher cell density and more uniform cell morphology. To further quantify cell growth, two indicators—cell number per 5 × 10^5^ μm^2^ (Fig. 5C) and average cell spreading area (Fig. 5D)—were analyzed. Both metrics confirmed that the HTO-LDH-PDA group supported superior cell attachment and spreading compared to the other two groups. The 3D dynamic videos showing BMSCs growing inside the Diamond sheet scaffolds are provided in Supporting Videos 1–3. Consistently, Cell Counting Kit-8 (CCK-8) assay results (Fig. 5B) showed that BMSCs in the HTO-LDH and HTO-LDH-PDA groups displayed significantly higher viability than those in the HTO group, indicating that LDH and PDA coatings have low cytotoxicity and high cytocompatibility. To visualize the detailed cell morphology, SEM imaging was performed after 7 days of culture (Fig. 5E). In the HTO group, most BMSCs appeared rounded or contracted, with limited pseudopod extension, suggesting suboptimal surface compatibility. In contrast, BMSCs in the LDH and PDA groups exhibited well-spread morphologies with firm adhesion, and the PDA group showed the highest cell density. To further elucidate the nanoscale interactions between BMSCs and the LDH nanostructures at the material interface, higher-magnification SEM images are provided in the Supporting Information (Fig. S8). These findings are consistent with the quantitative analyses and further support that PDA modification enhances cellular attachment and proliferation, thereby improving the overall cytocompatibility of the scaffold coatings.

Supplementary video related to this article can be found at https://doi.org/10.1016/j.bioactmat.2026.02.031

The following are the supplementary data related to this article:

Multimedia component 2 Multimedia component 2

Multimedia component 3 Multimedia component 3

Multimedia component 4 Multimedia component 4

Based on the results, the HTO coating exhibited significantly lower support for BMSCs adhesion and proliferation compared to the LDH and PDA groups, which may be attributed to differences in surface chemistry and interfacial properties. The HTO layer is primarily composed of Mg(OH)_2_ or MgO, featuring a hydrophobic surface that lacks polar functional groups. Such characteristics are unfavorable for the initial recognition and adhesion of cell membrane proteins. In contrast, the LDH coating possesses a layered nanostructure and abundant surface hydroxyl groups, which enhance hydrophilicity to some extent and promote cell attachment [39]. However, its biological functionality remains relatively limited. PDA coatings, on the other hand, are formed via the self-polymerization of dopamine, resulting in a surface enriched with catechol hydroxyl and amine groups [43]. These functional moieties facilitate hydrogen bonding and electrostatic interactions with polar groups on the cell membrane, while also improving protein adsorption—thereby creating a more bioactive interface that supports cell adhesion and spreading. Moreover, the superior wettability and interfacial energy modulation offered by PDA provide a more stable and favorable microenvironment for BMSCs, ultimately contributing to the significantly enhanced cytocompatibility observed in the PDA-modified group.

Beyond these general interfacial effects, each coating layer contributes a distinct and non-overlapping biological function. The HTO layer primarily serves as a corrosion-regulating barrier, ensuring controlled Mg^2+^ release and maintaining a relatively stable local ionic environment that is essential for cell survival. The LDH layer further enhances this effect by acting as an ion-buffering and protective intermediate, reducing excessive alkalization while providing a nano-textured surface that supports cell anchoring. Importantly, the PDA layer introduces a bioactive interface that actively promotes cell–material interactions through its abundant catechol and amine groups, which facilitate protein adsorption, focal adhesion formation, and extracellular matrix deposition. The synergistic integration of these layers therefore not only improves cytocompatibility but also establishes a biologically favorable microenvironment that supports sustained cell attachment, proliferation, and matrix remodeling, highlighting the biological superiority of the composite HTO–LDH–PDA coating over any single-layer modification.

To further verify the biological safety of Mg^2+^ toward BMSCs, flow cytometric analysis was performed to assess the expression of CD34, CD44, CD90, and CD29 (Fig. S9). The Blank group (no cells) showed no detectable signals. BMSCs exhibited a typical mesenchymal stem cell immunophenotype, with negative expression of CD34 and CD44 and positive expression of CD90 and CD29. Notably, BMSCs cultured in an Mg^2+^-enriched microenvironment for 24 h displayed an antigen expression pattern comparable to untreated BMSCs, indicating that Mg^2+^ exposure did not alter their characteristic surface marker profile. These results demonstrate that Mg^2+^ stimulation preserves the phenotypic stability and biological safety of BMSCs.

### 3.5 Regulation of the inflammatory microenvironment by Mg^2+^

To evaluate the immunomodulatory effects of Mg^2+^ on the inflammatory microenvironment, we examined the expression of two M1-specific pro-inflammatory markers—inducible nitric oxide synthase (iNOS) and tumor necrosis factor-alpha (TNF-α)—and two M2-specific anti-inflammatory markers—arginase-1 (Arg1) and interleukin-10 (IL-10)—at both the protein and mRNA levels. Five experimental groups were established: (1) NC group: RAW264.7 cells cultured under standard conditions; (2) M1 group: macrophages polarized to the M1 phenotype using Lipopolysaccharide (LPS) and Interferon gamma (IFN-γ); (3) M2 group: macrophages polarized to the M2 phenotype using Interleukin-4 (IL-4) and Interleukin-13 (IL-13); (4) Mg group: RAW264.7 cells cultured with Mg^2+^ extract; and (5) M1+Mg group: M1-polarized macrophages cultured with Mg^2+^ extract. All groups were cultured for 24 h before subsequent analysis. As shown in Fig. 5F, the expression of iNOS and TNF-α in the M1 group was significantly higher than in the other groups, confirming successful induction of the pro-inflammatory phenotype. Notably, while the M1+Mg group showed elevated expression of these markers compared to the Mg group, the levels were markedly lower than in the M1 group alone, suggesting that Mg^2+^ partially attenuated M1 polarization. Conversely, Arg1 and IL-10 were highly expressed in the M2 group, indicating effective induction of an anti-inflammatory phenotype. Interestingly, the M1+Mg group exhibited slightly reduced Arg1 and IL-10 expression compared to the Mg group but remained above the baseline level. These expression trends were consistently supported by densitometric analysis of Western blot bands (Fig. 5G) and qRT-PCR quantification (Fig. S4). These findings were further corroborated by flow cytometric analysis of macrophage phenotypic markers CD86 (M1) and CD206 (M2) at 24, 48, and 72 h (Fig. 5H). Consistent with the Western blot and qRT-PCR results, the M1 group exhibited sustained high CD86 expression over time, whereas Mg^2+^ treatment markedly reduced CD86 levels in the M1+Mg group in a time-dependent manner. In parallel, CD206 expression was significantly enhanced in the Mg group and moderately maintained in the M1+Mg group across all time points. Together, the phenotypic dynamics revealed by flow cytometry further confirm that Mg^2+^ modulates macrophage polarization by attenuating pro-inflammatory M1 characteristics while partially promoting an anti-inflammatory M2 phenotype.

In recent years, an increasing number of studies have highlighted the significant potential of Mg^2+^ in modulating the immune microenvironment, particularly in influencing macrophage polarization and alleviating inflammatory responses [44]. In this study, Mg^2+^ treatment markedly reduced the expression levels of M1-type pro-inflammatory markers, iNOS and TNF-α, suggesting its ability to inhibit pro-inflammatory polarization. Meanwhile, Mg^2+^ also exhibited a modulatory trend on M2-type markers, including Arg1 and IL-10, indicating its potential to promote immune phenotypic rebalancing under inflammatory conditions. Previous studies have suggested that Mg^2+^ can downregulate pro-inflammatory cytokine expression through multiple mechanisms, such as interfering with Toll-like receptor (TLR)-mediated inflammatory signaling pathways, reducing intracellular reactive oxygen species (ROS) generation, and stabilizing the cytoskeletal structure of immune cells [45]. This immunomodulatory capacity is of particular importance in the context of ischemic bone defect repair. By attenuating M1-type responses and enhancing immune tolerance, Mg^2+^ creates a more favorable local microenvironment for tissue regeneration, thereby facilitating angiogenesis, osteogenic differentiation, and scaffold integration.

### 3.6 Mg^2+^-induced osteoblastic differentiation and mineralization

**Fig. 6:**
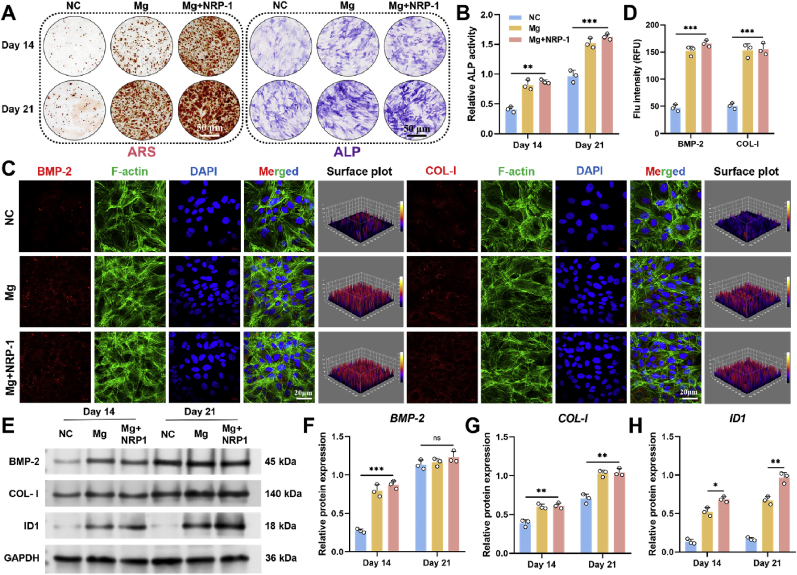
Mg^2+^-induced osteoblastic differentiation and mineralization. (**A**) ARS and ALP staining results of BMSCs cultured in three different microenvironments. (**B**) Quantitative analysis of relative ALP activity. Data are presented as mean values ± s.d. (n = 3). (**C**) Immunofluorescence staining of BMP-2 and COL-I, with DAPI for nuclear staining and F-actin for cytoskeleton labeling. (**D**) Quantitative analysis of Flu intensity. Data are presented as mean values ± s.d. (n = 3). (**E**) Western-blot analysis of BMP-2, COL-I, and ID1. (**F-H**) Quantitative analysis of relative protein expression of BMP-2, COL-I, and ID1. Data are presented as mean values ± s.d. (n = 3). ∗p < 0.05, ∗∗p < 0.01, ∗∗∗p < 0.001 (one-way ANOVA).

To evaluate the osteogenic potential of Mg^2+^ and its synergistic effect with NRP-1 on BMSCs, the experiment was divided into three groups: NC group, in which BMSCs were cultured in standard osteogenic induction medium; Mg group, with the addition of Mg^2+^ extract to the NC condition; and Mg + NRP-1 group, with 10 nM NRP-1 further added to the Mg group. As shown in Fig. 6A, both Alizarin Red S (ARS) and alkaline phosphatase (ALP) staining demonstrated that the Mg and Mg + NRP-1 groups exhibited significantly enhanced calcium deposition and early osteogenic activity compared to the NC group. Quantitative analysis of ALP activity (Fig. 6B) further confirmed that although there was no statistically significant difference between the Mg and Mg + NRP-1 groups, both showed markedly higher activity than the NC group, indicating that the addition of Mg^2+^ effectively promotes osteogenic differentiation of BMSCs. This trend was further corroborated by cetylpyridinium chloride (CPC)–based quantitative analysis of ARS staining, which revealed increased calcium deposition in the Mg-containing groups, consistent with the ALP activity results (Fig. S11).

To visualize the spatial distribution of osteogenesis-related markers within the cellular cytoskeleton, immunofluorescence staining was performed on day 21 using bone morphogenetic protein-2 (BMP-2) and type I collagen (COL-I) as representative indicators (Fig. 6C). The results revealed that both BMP-2 and COL-I signals were predominantly distributed around the nuclei in the Mg and Mg + NRP-1 groups, with markedly higher expression levels compared to the NC group. Further analysis using 3D surface plots and quantitative fluorescence intensity measurements (Fig. 6D) confirmed a significant upregulation of osteogenic marker expression in these two groups.

To more precisely assess the dynamic changes in osteogenesis-related proteins and genes during the induction process, Western-blot and qRT-PCR were employed to evaluate the expression levels of BMP-2, COL-I, and inhibitor of DNA binding 1 (ID1). As shown in Fig. 6E, the protein levels of BMP-2 and COL-I significantly increased at both day 14 and day 21, indicating a time-dependent enhancement in osteogenic activity. Although the band intensity of ID1 was relatively lower, its expression also exhibited an upward trend and showed a statistically significant difference compared to the NC group. These trends were further confirmed by densitometric analysis of the Western blot bands (Fig. 6F–H) and consistent qRT-PCR results (Fig. S5). Based on the transcriptomic enrichment analysis indicating the involvement of ID1-related signaling in the Mg^2+^/NRP-1–mediated osteogenic–angiogenic coupling, siRNA was employed to specifically suppress ID1 expression to further verify its functional role. qRT-PCR analysis confirmed effective siRNA-mediated knockdown of ID1. As shown in Fig. S13A, transfection with si-ID1 resulted in a significant reduction in ID1 mRNA expression compared with the si-NC group. Western-blot analysis confirmed that ID1 protein levels were markedly reduced following siRNA transfection compared with the Mg^2+^ + NRP-1 control group (Fig. S14). Importantly, ID1 knockdown significantly attenuated the activation of downstream osteogenic signaling induced by Mg^2+^/NRP-1 stimulation, indicating that the regulatory effects of Mg^2+^/NRP-1 on the osteogenic–angiogenic axis are, at least in part, dependent on ID1. These findings provide direct mechanistic evidence that ID1 acts as a critical intermediate target in Mg^2+^/NRP-1–driven signaling.

Collectively, these results demonstrate that Mg^2+^ significantly enhances the osteogenic differentiation of BMSCs. Notably, the early-stage upregulation of Inhibitor of ID1 suggests its potential regulatory role in Mg^2+^-mediated osteogenesis. Previous studies have identified ID1 as a downstream target of the TGF-β/SMAD signaling pathway, which can promote osteogenesis by suppressing osteogenic inhibitors and synergizing with BMP-2 to induce bone formation [46]. Furthermore, BMP-2, as a well-established osteoinductive factor, activates SMAD1/5/8 and initiates transcription of downstream bone matrix proteins such as COL-I [47]. Based on these findings, we hypothesize that Mg^2+^ may activate SMAD signaling mediated by TGF-β family members, leading to the upregulation of ID1, which in turn enhances the expression of BMP-2 and its downstream effector COL-I. This cascade may constitute an “ID1–BMP-2–COL-I″ axis that ultimately promotes the osteogenic differentiation of BMSCs.

### 3.7 Evaluation of angiogenic potential induced by Mg^2+^ and NRP-1

**Fig. 7:**
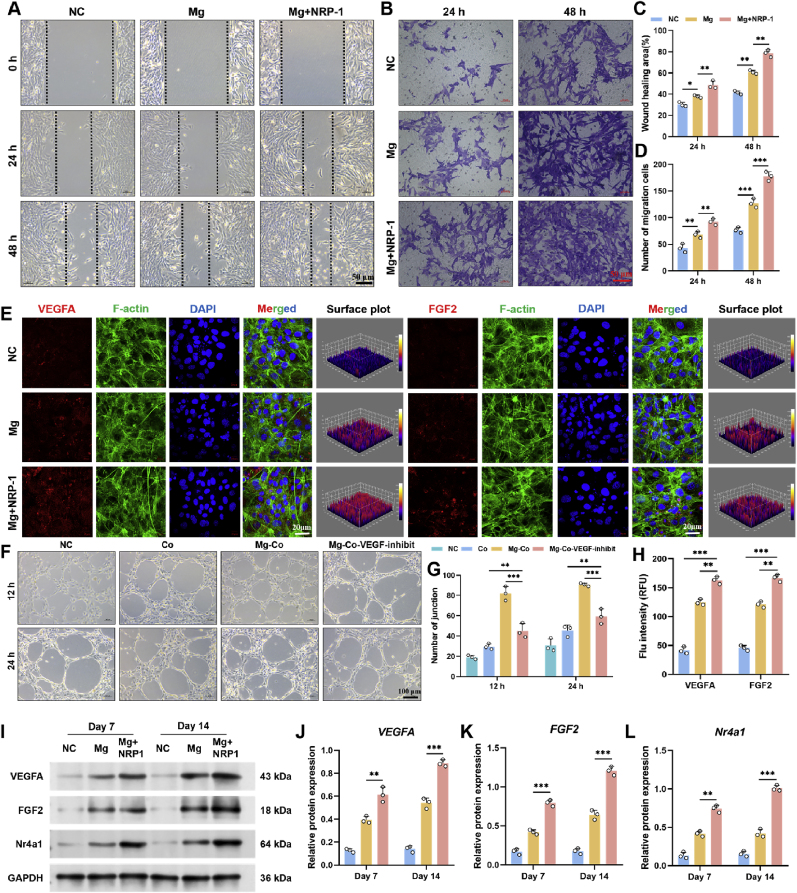
Evaluation of angiogenic potential induced by Mg^2+^ and NRP-1. (**A**) Scratch assay in three different microenvironments. (**B**) Transwell assay in three different microenvironments. (**C**) Quantitative analysis of wound healing area. (**D**) Quantitative analysis of number of migration cells. (**E**) Immunofluorescence staining of VEGFA and FGF2, with DAPI for nuclear staining and F-actin for cytoskeleton labeling. (**F**) Tube formation evaluation in four different microenvironments. (**G**) Quantitative analysis of number of junction. (**H**) Quantitative analysis of Flu intensity. (**I**) Western-blot analysis of VEGFA, FGF2, and Nr4a1. (**J-L**) Quantitative analysis of relative protein expression of VEGFA, FGF2, and Nr4a1. Data are presented as mean values ± s.d. (n = 3). ∗p < 0.05, ∗∗p < 0.01, ∗∗∗p < 0.001 (one-way ANOVA).

To evaluate the effects of Mg^2+^ and its synergistic interaction with NRP-1 on BMSC migration, scratch assay (Fig. 7A) and transwell assay (Fig. 7B) were performed across three experimental groups. The results showed that the Mg + NRP-1 group exhibited the most pronounced enhancement in both wound closure area and the number of migrated cells through the transwell membrane, followed by the Mg group, which was also significantly higher than the NC group. Quantitative analysis of the wound healing area (Fig. 7C) and the number of migrated cells (Fig. 7D) further confirmed these trends. To gain deeper insight into cell–cell communication, we redesigned the tube formation assay with an optimized grouping strategy. Four groups were established: (i) NC, Human Umbilical Vein Endothelial Cells (HUVECs) cultured alone; (ii) Co, co-culture of HUVECs and BMSCs; (iii) Mg + Co, co-culture of HUVECs with BMSCs pretreated with Mg^2+^ extract for 24 h; and (iv) Mg + Co + VEGF + inhibit, co-culture of HUVECs with Mg^2+^-pretreated BMSCs in the presence of a VEGFA inhibitor. Tube formation was evaluated at two time points (12 and 24 h). The results demonstrated that BMSCs preconditioned with Mg^2+^ markedly enhanced the angiogenic capacity of HUVECs, as evidenced by more extensive and well-organized capillary-like networks (Fig. 7F). In contrast, the addition of a VEGFA inhibitor significantly attenuated this pro-angiogenic effect. These findings and quantification (Fig. 7G) indicate that Mg^2+^ stimulation upregulates VEGF secretion from BMSCs, which serves as a key paracrine mediator driving HUVECs angiogenesis, thereby highlighting the critical role of Mg^2+^-modulated cell–cell communication in regulating vascular formation.

To visualize the spatial distribution of angiogenesis-related genes within the cytoskeletal framework, VEGFA and fibroblast growth factor 2 (FGF2) were selected as representative markers and assessed via immunofluorescence staining on day 14 (Fig. 7E). The results showed only minimal positive expression in the NC group, while the Mg group exhibited a noticeable increase in VEGFA and FGF2 signals. Notably, the Mg + NRP-1 group demonstrated the strongest fluorescent intensity, primarily localized around the perinuclear region. Further analysis using ImageJ-based 3D surface plotting and fluorescence intensity quantification (Fig. 7H) revealed a clear upward trend across the three groups, with the Mg + NRP-1 group showing significantly higher expression levels than both the Mg and NC groups. These findings indicate that the combination of Mg^2+^ and NRP-1 markedly enhances the expression of angiogenesis-related genes.

To further elucidate the dynamic changes in angiogenic protein and gene expression during induction, Western-blot and qRT-PCR analyses were performed to evaluate the expression levels of VEGFA, FGF2, and the nuclear receptor subfamily 4 group A member 1 (Nr4a1). As shown in Fig. 7I, the Mg group exhibited a moderate upregulation of these markers at both day 7 and day 14, whereas the Mg + NRP-1 group demonstrated significantly higher expression levels of VEGFA, FGF2, and Nr4a1 at both time points compared to the other groups, with statistical significance. This expression pattern was consistently confirmed by grayscale quantification of the Western blot bands (Fig. 7J–L) and qRT-PCR results (Fig. S6), further supporting that the synergistic action of Mg^2+^ and NRP-1 can significantly enhance the angiogenic potential of BMSCs. Similarly, to validate the involvement of Nr4a1 identified from pathway enrichment analysis, siRNA-mediated knockdown of Nr4a1 was performed. NR4A1 mRNA levels were markedly decreased in cells transfected with si-Nr4a1, as shown in Fig. S13B. Western-blot results demonstrated efficient suppression of Nr4a1 expression in the siRNA-treated group compared with the Mg^2+^ + NRP-1 control group (Fig. S16). Notably, inhibition of Nr4a1 substantially weakened the downstream signaling responses associated with angiogenic regulation under Mg^2+^/NRP-1 stimulation. These results indicate that Nr4a1 functions as another essential mediator in the Mg^2+^/NRP-1–regulated signaling network.

NRP-1 is broadly expressed in endothelial cells, osteoblasts, and various stem cells, and has garnered significant attention due to its critical roles in angiogenesis, cell migration, and tissue regeneration [48]. As a co-receptor of VEGF, NRP-1 can markedly enhance VEGF/VEGFR-mediated signal transduction. In this study, it notably augmented BMSC migration and capillary-like structure formation. Furthermore, the expression levels of VEGFA, FGF2, and Nr4a1 were significantly upregulated under NRP-1 treatment, suggesting the presence of a synergistically regulated angiogenic signaling axis. Previous studies have reported that FGF2 promotes endothelial cell proliferation and survival via activation of the PI3K-AKT pathway, while Nr4a1, as a downstream responsive gene of AKT signaling, contributes to vascular homeostasis [49]. Consistent with these findings, our results demonstrated a sequential upregulation of these factors, indicating that VEGFA–FGF2–Nr4a1 may constitute an angiogenic axis potentially linked to the PI3K-AKT pathway. This axis, enhanced by NRP-1, may collectively mediate the observed pro-angiogenic effects.

### 3.8 Postoperative observation of ischemic bone defect repair

**Fig. 8:**
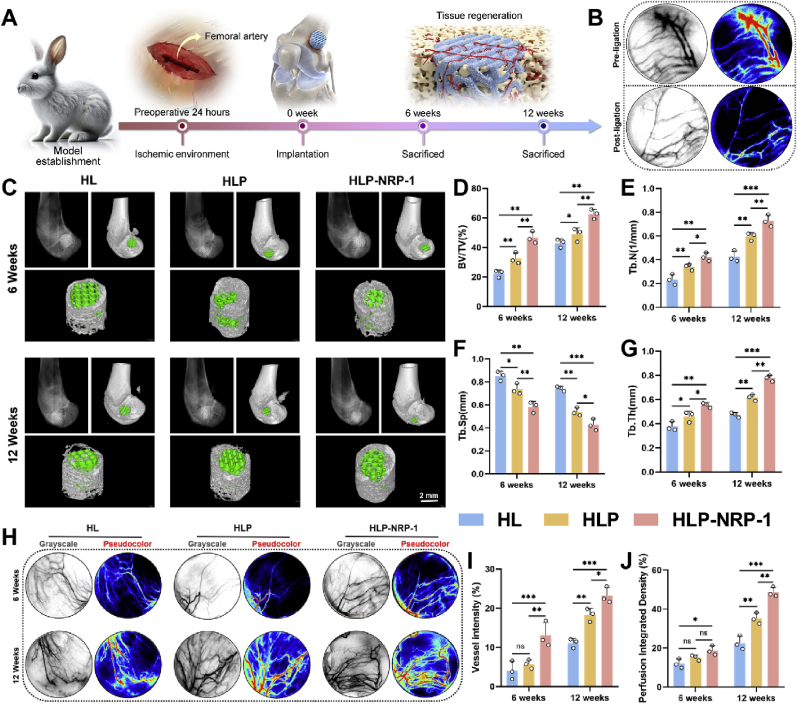
Imaging evaluation in animal experiments. (**A**) Schematic diagram of animal experiments. (**B**) Pre- and postoperative speckle contrast imaging of the periosteum at the lateral femoral condyle. (**C**) X-ray and CT images of the bone defect repair site at 6 and 12 weeks postoperatively (green indicates the implanted scaffold; gray indicates the surrounding newly formed bone). (**D-G**) Quantitative analysis of BV/TV, Tb.N, Tb. Sp, Tb.Th. Data are presented as mean values ± s.d. (n = 3). (**H**) Laser speckle contrast images of the bone defect repair site in different groups at 6 and 12 weeks postoperatively. (**I-J**) Quantitative analysis of vessel intensity and perfusion integrated density. Data are presented as mean values ± s.d. (n = 3). ns, no significant (p > 0.05), ∗p < 0.05, ∗∗p < 0.01, ∗∗∗p < 0.001 (one-way ANOVA).

This study utilized 30 female New Zealand rabbits weighing 2.0–2.5 kg as *in vivo* experimental models, which were randomly divided into three groups: the HL group implanted with HTO-LDH coated Mg scaffolds; the HLP group implanted with HTO-LDH-PDA coated Mg scaffolds; and the HLP-NRP-1 group implanted with HTO-LDH-PDA coated scaffolds pre-soaked in 10 nM NRP-1-loaded lipos solution for 45 min before implantation. As shown in Fig. 8A, all animals underwent ligation of the femoral artery on the operative side 24 h prior to the main surgery. Laser speckle contrast imaging performed before and after the procedure (Fig. 8B) demonstrated a significant reduction in blood flow in the lateral femoral condyle region. Notably, none of the 30 animals exhibited signs of ischemic necrosis in the affected limb during the two-week postoperative observation period, confirming the successful establishment of the ischemic bone defect model.

As shown in Fig. 8C, X-ray imaging revealed that the implanted scaffolds in all groups maintained relatively intact structural integrity, with no obvious gaps between the scaffold and surrounding bone tissue in the defect area, indicating favorable osseointegration. Surface reconstruction images from micro-CT further illustrated the spatial distribution of newly formed bone within the defect region, showing a progressive increase in bone formation over time and with more advanced treatment. Given that the defect and scaffold were both cylindrical in shape with a diameter and height of 5 mm, the region of interest (ROI) for micro-CT analysis was defined as a cylinder centered on the scaffold with a diameter and height of 5.5 mm to more precisely evaluate new bone ingrowth around the scaffold. The results demonstrated that the HLP-NRP-1 group exhibited denser new bone formation within the defect region at both 6 and 12 weeks post-implantation. Quantitative analysis of four key microstructural parameters—bone volume fraction (BV/TV), trabecular number (Tb.N), trabecular separation (Tb.Sp), and trabecular thickness (Tb.Th) (Fig. 8D–G)—further confirmed that the HLP-NRP-1 group achieved superior bone regeneration compared to the other groups, suggesting enhanced osteogenic activity associated with this treatment.

Neovascularization within the defect region is also a critical indicator in the bone repair process. To assess the angiogenic response across different treatment groups, laser speckle contrast imaging was employed to evaluate local blood perfusion in the defect areas of rabbit femoral condyles. As shown in Fig. 8H, among the HL and HLP groups, only the HLP group demonstrated relatively evident intrabony neovascularization at 12 weeks postoperatively. In contrast, the HLP-NRP-1 group exhibited markedly enhanced vascular structures at both 6 and 12 weeks, suggesting a superior pro-angiogenic capacity. To objectively quantify these observations, vessel intensity (Fig. 8I) and perfusion integrated density (Fig. 8J) were measured. The results were consistent with the qualitative assessments, indicating that the HLP-NRP-1 group achieved the most robust vascular regeneration among all groups, thereby highlighting its enhanced potential for promoting neovascularization in ischemic bone defects.

In this study, Mg scaffolds demonstrated excellent cytocompatibility and bone integration in a rabbit model of ischemic bone defect, highlighting their significant osteogenic potential [50]. However, despite all groups receiving structurally comparable Mg-based implants, differences in bone regeneration were still observed at various postoperative time points. This suggests that, in addition to the material properties, the local microenvironment plays a pivotal role in regulating the bone healing process. Bone regeneration is a dynamic process that heavily relies on vascular support, especially in ischemic defect regions where nutrient and oxygen supply is inherently compromised [51,52]. In such contexts, neovascularization becomes essential for maintaining cellular activity and promoting new bone formation. Notably, angiogenesis and osteogenesis are highly interdependent processes. According to the results of laser speckle contrast imaging and micro-CT analysis, the HLP-NRP-1 group exhibited markedly enhanced vascularization, which was accompanied by denser bone formation at both 6 and 12 weeks post-surgery compared to the other groups. These findings indicate that improved vascular regeneration may provide a more favorable metabolic microenvironment for bone tissue repair, thereby contributing to superior overall healing outcomes.

### 3.9 Histological evaluation of bone regeneration and angiogenesis

**Fig. 9:**
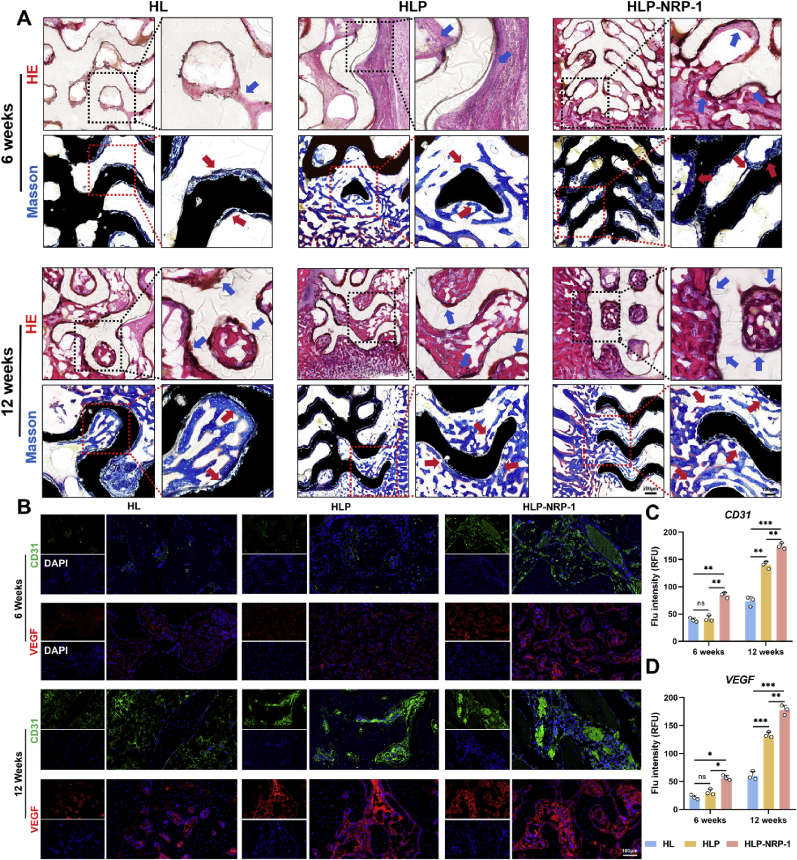
Histological staining and immunofluorescence analysis. (**A**) HE and Masson staining of hard tissue sections at 6 and 12 weeks. The blue arrows indicate newly formed connective or osteogenic tissue, while the red arrows indicate newly deposited collagen fibers. (**B**) Immunofluorescence staining of CD31 and VEGF at 6 and 12 weeks. Nuclei were counterstained with DAPI. (**C-D**) Quantitative analysis of Flu intensity of CD31 and VEGF. Data are presented as mean values ± s.d. (n = 3). ns, no significant (p > 0.05), ∗p < 0.05, ∗∗p < 0.01, ∗∗∗p < 0.001 (one-way ANOVA).

To evaluate the *in vivo* bone repair performance of the implanted scaffolds in each group, histological analyses using hematoxylin and eosin (HE) staining and Masson's trichrome staining were performed (Fig. 9A). The results showed that all three groups of Mg alloy scaffolds maintained relatively intact structural integrity after implantation. At 6 weeks, only a small amount of newly formed connective tissue and collagen fibers was observed around the scaffolds in the HL and HLP groups, whereas the HLP-NRP-1 group exhibited markedly increased tissue attachment. By 12 weeks, all groups showed enhanced tissue coverage around the scaffolds, however, the HLP-NRP-1 group demonstrated superior outcomes in terms of the quantity, organization, and density of newly formed connective tissue, osteoid, and collagen fibers, indicating its enhanced capacity to promote tissue integration and bone regeneration.

To further evaluate neovascularization in the bone defect repair area, CD31 and VEGF immunofluorescence staining was performed as vascularization markers (Fig. 9B). The results showed that in the HL group, only sparse positive vascular signals were observed at both 6 and 12 weeks postoperatively. In the HLP group, locally increased neovascular expression was noted at 12 weeks, but the vascular density and distribution remained significantly different from those of normal tissue. In contrast, the HLP-NRP-1 group exhibited the most favorable outcomes in terms of vessel density, diameter, and spatial distribution, indicating a superior capacity for vascular regeneration. Quantitative analysis of CD31 (Fig. 9C) and VEGF (Fig. 9D) fluorescence intensity further confirmed the significant enhancement of neovascularization in the HLP-NRP-1 group, underscoring its unique proangiogenic effect within the ischemic bone defect microenvironment.

In bone tissue engineering, establishing a microenvironment with robust vascularization potential is widely recognized as a critical factor for enhancing the quality of bone regeneration [53,54]. In this study, the HLP-NRP-1 group demonstrated significant advantages in both tissue structural reconstruction and neovascularization, suggesting that the local level of vascularization largely determines the outcome of bone repair. Loading NRP-1 onto the scaffold surface markedly increased the density and spatial integrity of new blood vessels within the defect area. Enhanced angiogenic capacity not only accelerated the formation rate of new bone tissue but also promoted alignment and structural density of the regenerated tissue, closely resembling that of the surrounding native bone, thereby achieving coordinated restoration of both function and architecture. These findings indicate that the NRP-1-mediated optimization of the vascular microenvironment extends beyond simply augmenting angiogenesis; it facilitates the coupling between angiogenesis and osteogenesis, thereby indirectly improving overall bone regeneration quality [55]. To further evaluate the systemic biological safety of Mg^2+^, histological examination of major organs, including the liver, spleen, kidney, and heart, was performed. As shown in Fig. S21, no evident pathological abnormalities, inflammatory infiltration, or tissue damage were observed in these organs, indicating that implantation of the Mg-based scaffold did not induce detectable biological toxicity or adverse effects on vital organ function. Our results further corroborate the intimate biological interplay between vascularization and osteogenesis, emphasizing that the synergistic enhancement of both processes is essential for high-quality tissue reconstruction in ischemic bone defect repair.

### 3.10 Transcriptomic analysis and pathway validation

**Fig. 10:**
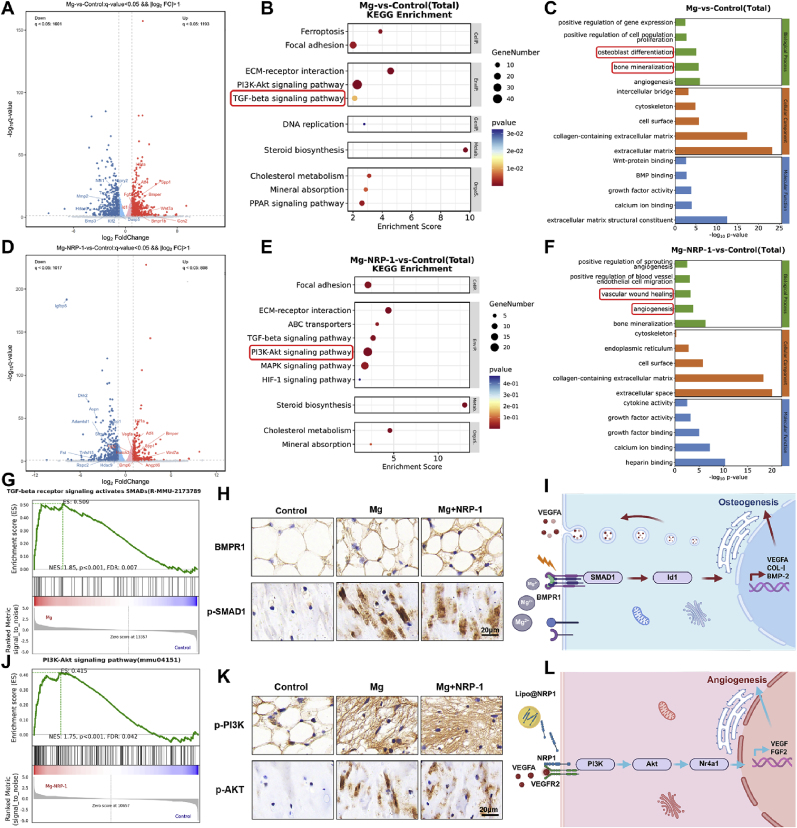
Transcriptomic analysis and pathway validation. (**A**) Volcanic enrichment map of transcriptomic sequencing of BMSCs treated with Mg^2+^. (**B**) KEGG enrichment analysis of BMSCs treated with Mg^2+^. (**C**) GO enrichment analysis of BMSCs treated with Mg^2+^. (**D**) Volcanic enrichment map of BMSCs treated with Mg-NRP-1. (**E**) KEGG enrichment analysis of BMSCs treated with Mg-NRP-1. (**F**) GO enrichment analysis of BMSCs treated with Mg-NRP-1. (**G**) GSEA analysis of the TGF-β/SMAD signaling pathway (FDR: 0.007). (**H**) Immunohistochemical analysis of BMPR1 and p-SMAD1. (**I**) Hypothetical schematic of Mg-mediated osteogenic signaling pathways. (**J**) GSEA analysis of the PI3K/AKT signaling pathway (FDR: 0.042). (**K**) Immunohistochemical analysis of p-PI3K and p-AKT. (**L**) Hypothetical schematic of Mg-NRP-1-mediated angiogenic signaling pathways.

To further elucidate the regulatory mechanisms by which Mg influences osteogenic differentiation of BMSCs, RNA transcriptome sequencing was performed to compare the NC and Mg groups. The volcano plot (Fig. 10A) revealed a large number of significantly upregulated and downregulated genes in the Mg group. Kyoto encyclopedia of genes and genomes (KEGG) enrichment analysis (Fig. 10B) identified a prominent enrichment of the TGF-β signaling pathway, while gene ontology (GO) analysis (Fig. 10C) further indicated significant enrichment in osteogenesis-related biological processes such as "osteoblast differentiation" and "bone mineralization." To validate the activation of this osteogenic signaling pathway, gene set enrichment analysis (GSEA) analysis was specifically conducted for the TGF-β pathway (Fig. 10G), which showed a marked upregulation in the Mg group. Based on these findings, two key targets within the pathway, bone morphogenetic protein receptor type 1 (BMPR1) and phosphorylated SMAD1/5/8 (p-SMAD), were selected for immunohistochemical validation (Fig. 10H). Notably, both markers exhibited higher positive expression in the Mg alloy scaffold, further supporting the involvement of the TGF-β/SMAD pathway in Mg-mediated osteogenic regulation (Fig. 10I).

To further investigate the regulatory mechanism by which NRP-1 combined with Mg influences the angiogenic potential of BMSCs, RNA transcriptome sequencing was performed to compare the NC group and the Mg + NRP-1 group. The volcano plot (Fig. 10D) revealed a large number of differentially expressed genes. KEGG enrichment analysis (Fig. 10E) identified significant enrichment of the PI3K-AKT signaling pathway, while GO analysis (Fig. 10F) showed enrichment in vascular-related biological processes, including “vascular wound healing” and “angiogenesis.” To further validate the activation of this pathway, GSEA analysis was conducted specifically on the PI3K-AKT pathway (Fig. 10J), which demonstrated a significant upregulation in the Mg + NRP-1 group. Based on these findings, two key downstream targets—phosphorylated PI3K (p-PI3K) and phosphorylated AKT (p-AKT)—were selected for immunohistochemical evaluation (Fig. 10K). The results revealed markedly higher positive expression levels in the Mg + NRP-1 group compared to the other groups, further confirming the pivotal role of the PI3K-AKT pathway in mediating the pro-angiogenic effect of Mg in combination with NRP-1 (Fig. 10L).

Although this study provides preliminary evidence supporting the proposed mechanism, several limitations remain. For instance, it is still unclear whether direct crosstalk exists between the TGF-β/SMAD and PI3K-AKT signaling pathways, whether key molecular components act in a hierarchical or sequential regulatory manner, and whether this coupling mechanism is universally applicable at the cellular level. These unresolved issues suggest that future studies should focus on identifying potential interaction nodes between the pathways, aiming to elucidate a more comprehensive mechanism underlying bone–vascular co-regeneration. Such insights could offer a stronger theoretical basis for developing time-sensitive and targeted strategies in personalized bone repair.

## 4 Conclusion

This study hierarchically constructed Mg alloy porous scaffolds with composite coatings, which enabled the regulated release of Mg ions and NRP-1 for the treatment of ischemic bone defects. The biodegradable Mg alloy scaffolds were printed in customized shapes and with tailored pores, providing reliable mechanical support and proper space for bone in-growth. The composite coatings integrated HTO, LDH, and PDA layers, and endowed the scaffolds with controlled degradation and structural integrity during implantation. The favorably released Mg ions enhanced biocompatibility, modulated inflammatory reactions, promoted osteoblastic differentiation and mineralization, and improved angiogenic potential, as evidenced by in vitro evaluations. The bioactive protein NRP-1 was successfully encapsulated into liposomes, referred as NRP-1-loaded lipos, and was then effectively loaded onto the PDA layer. The synergistic release of Mg ions and NRP-1 further significantly enhanced the vascularization process.

In the developed animal model of ischemic bone defects, this composite scaffold demonstrated improved vascular regeneration and new bone formation. Multidimensional analyses, including histology, imaging, and molecular mechanisms, consistently indicated that the scaffold significantly improved bone regeneration quality by synergistically regulating the vascular-bone microenvironment via the TGF-β/SMAD and PI3K-AKT signaling pathways. Overall, this work innovatively proposes the "Mg–NRP-1 synergistic axis" strategy for coupling angiogenesis and osteogenesis, offering a novel approach to addressing challenges in complex bone defect repair. Future studies can further investigate the regulatory networks and spatiotemporal dynamics between these signaling pathways to advance the development of controllable and precision-engineered bone tissue scaffolds.

## CRediT authorship contribution statement

**Zijie Pei:** Writing – review & editing, Writing – original draft, Project administration. **Haojing Xu:** Writing – review & editing, Writing – original draft, Investigation. **Piqian Zhao:** Methodology, Investigation. **Ya Wen:** Formal analysis. **Ze Zhang:** Formal analysis. **Liangkun Huang:** Software, Methodology. **Mengyu Wang:** Conceptualization. **Bo Peng:** Data curation. **Liangyuan Wen:** Supervision, Funding acquisition. **Peng Wen:** Supervision, Funding acquisition. **Fengpo Sun:** Supervision, Funding acquisition.

## Ethics approval and consent to participate

All animal experiments were conducted in strict compliance with the Animal Care and Use Committee guidelines, adhering to the principles outlined in the Guide for the Care and Use of Laboratory Animals by the National Institutes of Health, USA, and the animal experiment protocol was approved by the laboratory animal welfare ethics committee of the China Inspection Huatongwei Testing Co. (Animal Ethics review number: QRB219-21).

## Declaration of competing interest

Peng Wen is an editorial board member for Bioactive Materials and was not involved in the editorial review or the decision to publish this article. All authors declare that there are no competing interests.
