# Supplementary material for: Reconstructing the ischemic osteogenic microenvironment through hierarchical scaffolds orchestrating Mg2+ signaling and neuropilin-1–mediated angiogenesis
Source: Bioact Mater. 2026 Mar 26;62:738–58. doi: 10.1016/j.bioactmat.2026.02.031 (PMC13059125; doi:10.1016/j.bioactmat.2026.02.031)
Supplement: Multimedia component 1 [file mmc1.docx]

**Supporting Information**

**Reconstructing the Ischemic Osteogenic Microenvironment through Hierarchical Scaffolds Orchestrating Mg²⁺ Signaling and Neuropilin-1–Mediated Angiogenesis**

**Table S1.** Main elemental composition (at. %) of selected points in Figure. 2A and the as-printed scaffolds surface measured by EDX

|  | Mg | Y | Nd | O | Al | N |
| --- | --- | --- | --- | --- | --- | --- |
| Point 01 | 28.7±1.5 | 7.1±1.1 | 9.8±2.4 | 54.2±3.3 | / | / |
| Point 02 | 29.5±2.7 | 5.9±0.7 | 10.0±2.6 | 52.3±0.4 | 2.0±0.1 | / |
| Point 03 | 27.3±2.1 | 6.0±0.4 | 10.4±1.5 | 49.2±2.6 | 1.3±0.2 | 5.1±0.3 |
| As-printed scaffold | 95.6±0.3 | 0.8±0.1 | 0.5±0.1 | 0.5±0.1 | / | / |

**Table S2.** Fitted EIS results of different samples based on the corresponding equivalent circuit models

|  | HTO | HTO-LDH | HTO-LDH-PDA |
| --- | --- | --- | --- |
| R_s_ (Ω·cm^2^) | 7.00 | 9.48 | 8.83 |
| CPE_1_ (Ω^-1^·cm^-2^) | 7.85*10^-6^ | 3.38*10^-5^ | 2.14*10^-5^ |
| n_1_ | 0.84 | 0.58 | 0.70 |
| R_1_ (Ω·cm^2^)  CPE_2_ (Ω^-1^·cm^-2^)  n_2_  R_2_ (Ω·cm^2^) | 294  1.14*10^-4^  0.98  120 | 4151  7.50*10^-4^  0.78  4194 | 6381  6.65*10^-4^  0.80  5488 |

**Table. S3.** Primers related to qRT-PCR for inflammation regulation validation

| Gene | 5’-3’ Sequence |
| --- | --- |
| β-actin-F: | AGCCATGTACGTAGCCATCC |
| β-actin-R: | CTCTCAGCTGTGGTGGTGAA |
| iNOS-F: | GGAATGGGCTCCTGACAAGT |
| iNOS-R: | TAGAAAGGGGGGTCGGTTTC |
| TNF-α-F: | TGAGAGGGAGAGGAGGAGAAC |
| TNF-α-R: | GAGGCCATTTGGGAACTTG |
| Arg1-F: | CTGGGGAAGGTGTTTGTCAG |
| Arg1-R: | CAGGAAGGCTTTCAGGTCAG |
| IL-10-F: | CTGCTGGTACTGCTGAGATG |
| IL-10-R: | GCTCTTGGTCTTGGTTCTTGG |

**Table. S4.** Primers related to qRT-PCR for osteoblast regulation validation

| Gene | 5’-3’ Sequence |
| --- | --- |
| GAPDH-F: | ACCCAGAAGACTGTGGATGG |
| GAPDH-R: | ACACATTGGGGGTAGGAACA |
| BMP-2-F: | CAAACGAGAAAAGCGTCAAGC |
| BMP-2-R: | ATCCAGTCATTCCACCCCACA |
| COL-I-F: | GCACGAGTCACACCGGAACT |
| COL-I-R: | TCAGCAGGACCAGGAAGACC |
| ID1-F: | TGAGCAGGTGAGCACGTAAG |
| ID1-R: | GAGTCCCTGATGTAGTCGTAG |

**Table. S5.** Primers related to qRT-PCR for angiogenesis regulation validation

| Gene | 5’-3’ Sequence |
| --- | --- |
| GAPDH-F: | ACCCAGAAGACTGTGGATGG |
| GAPDH-R: | ACACATTGGGGGTAGGAACA |
| VEGFA-F: | GCTCTCTTTCTGCCCCAG |
| VEGFA-R: | TGGTTGAGGGTCTCGATT |
| FGF2-F: | AAGCGGCTCTACTGCAAGAAC |
| FGF2-R: | CCTTGATAGACACAACTCCTCTC |
| Nr4a1-F: | GGACAACGCTTCATGCCAGCAT |
| Nr4a1-R: | CCTTGTTAGCCAGGCAGATGTAC |


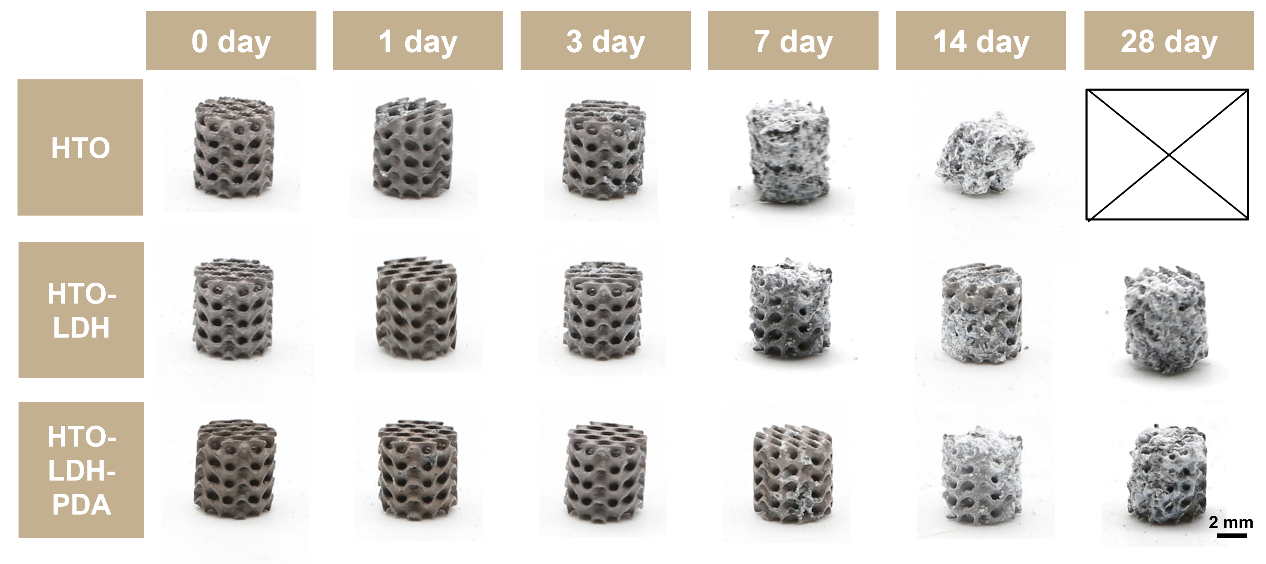


**Fig. S1.** Morphology of HTO, HTO-LDH, and HTO-LDH-PDA scaffolds after corrosion in Hank's simulated body fluid.


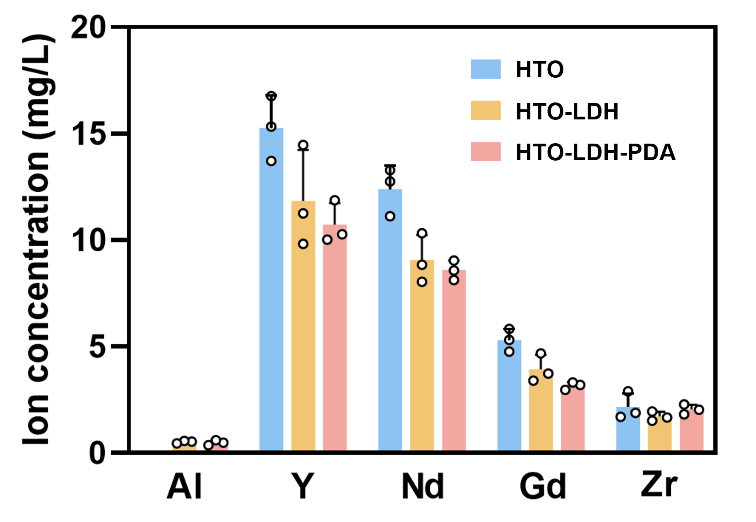


**Fig. S2.** Other elements release from HTO, HTO-LDH and HTO-LDH-PDA scaffolds after 28 days in vitro immersion.


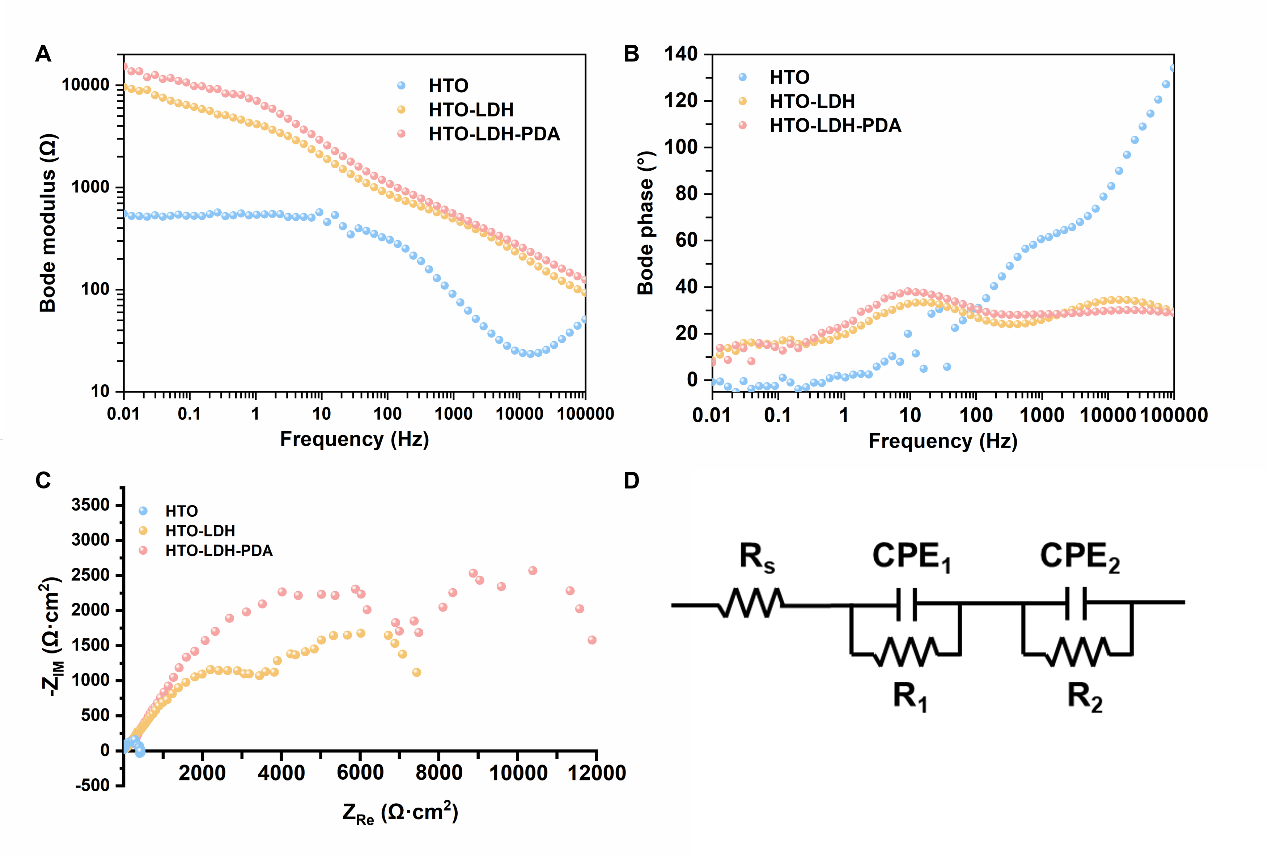


**Figure S3.** Electrochemical impedance spectroscopy results. (**A**) Bode modulus. (**B**) Bode phase. **(C)** Nyquist plots. **(D)** equivalent circuits of the samples


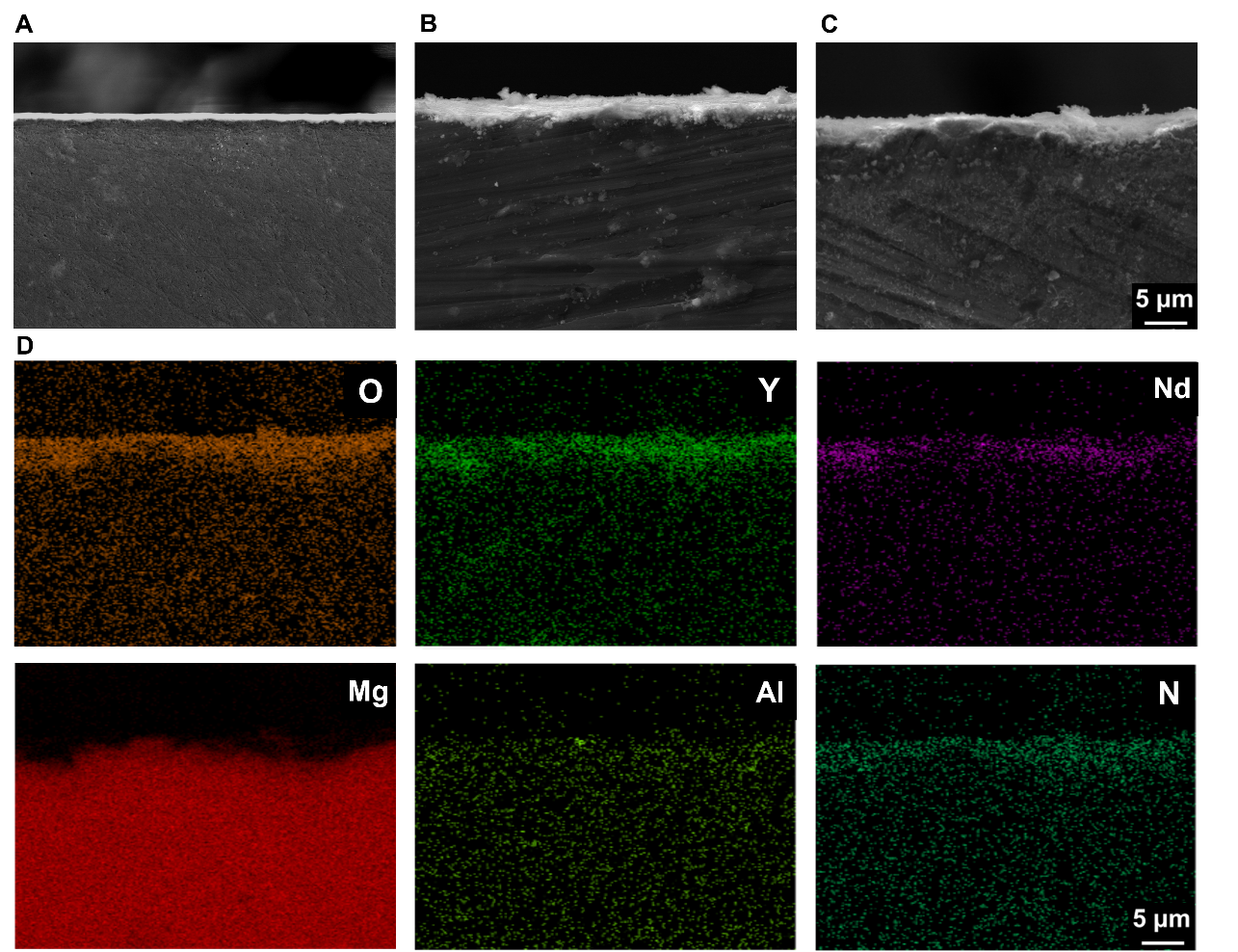


**Figure S4.** Cross-sectional SEM image of (A) HTO (B) HTO-LDH (C) HTO-LDH-PDA scaffolds. (D) EDX mapping of HTO-LDH-PDA cross section.

**
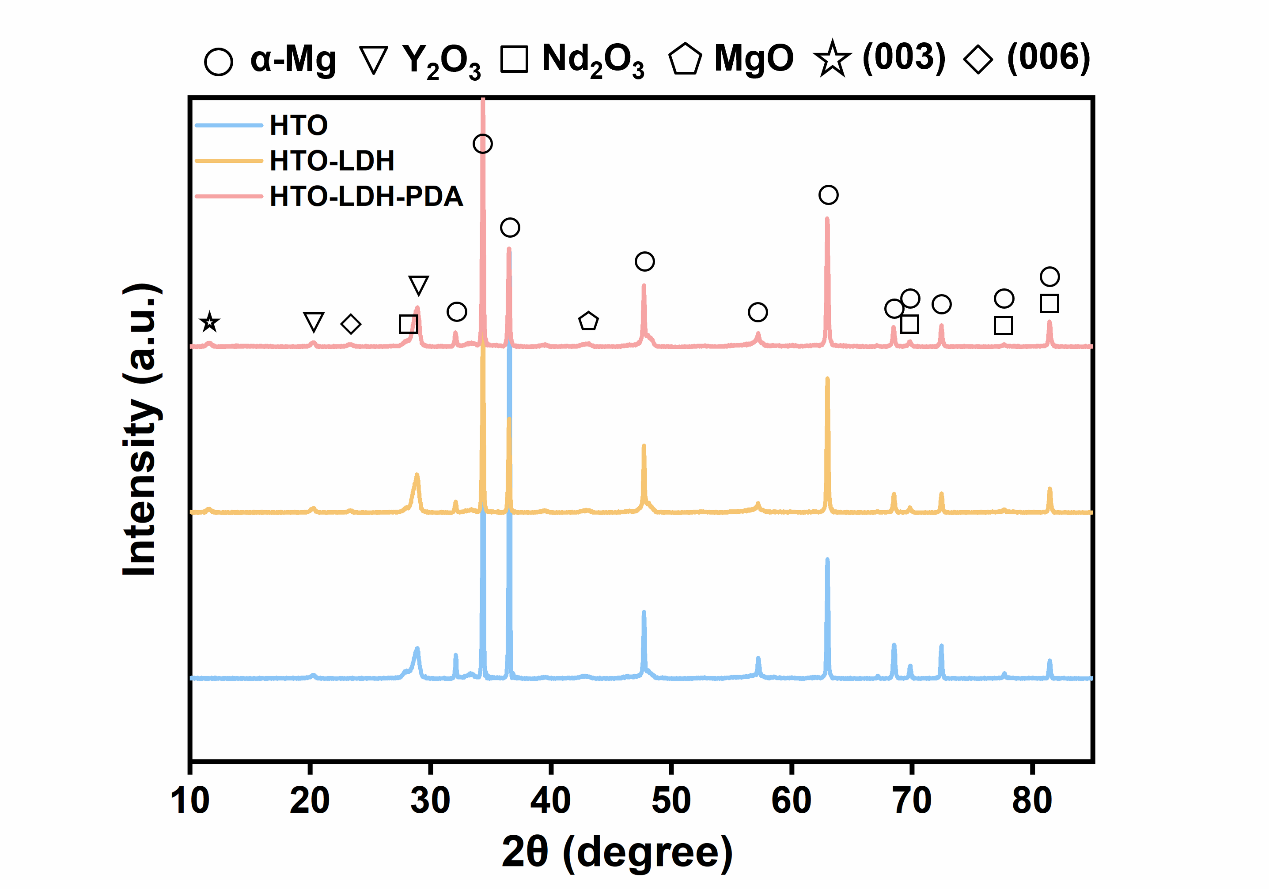
**

**Figure S5.** XRD patterns of the scaffolds with different coatings


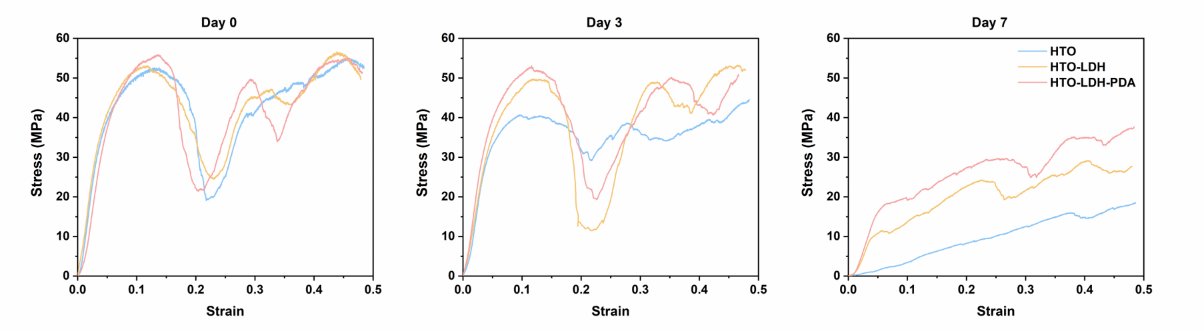


**Figure S6.** Compressive stress-strain (σ-ε) curves of scaffolds at day 0, 3 and 7


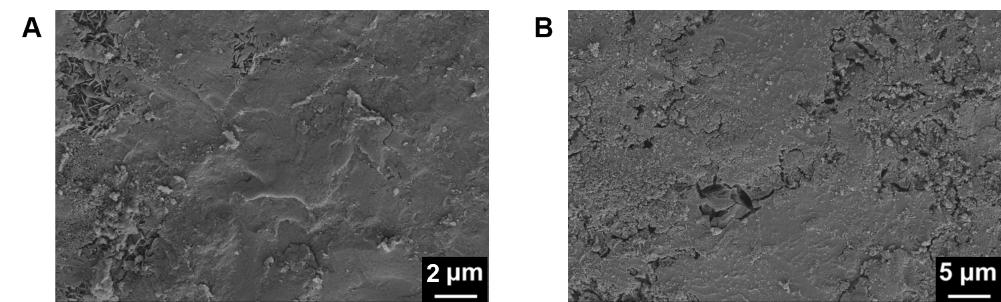


**Figure S7.** Surface morphology of (A) the Mg scaffold with LDH directly coated without oxidation and (B) the HTO scaffold with PDA directly attached.


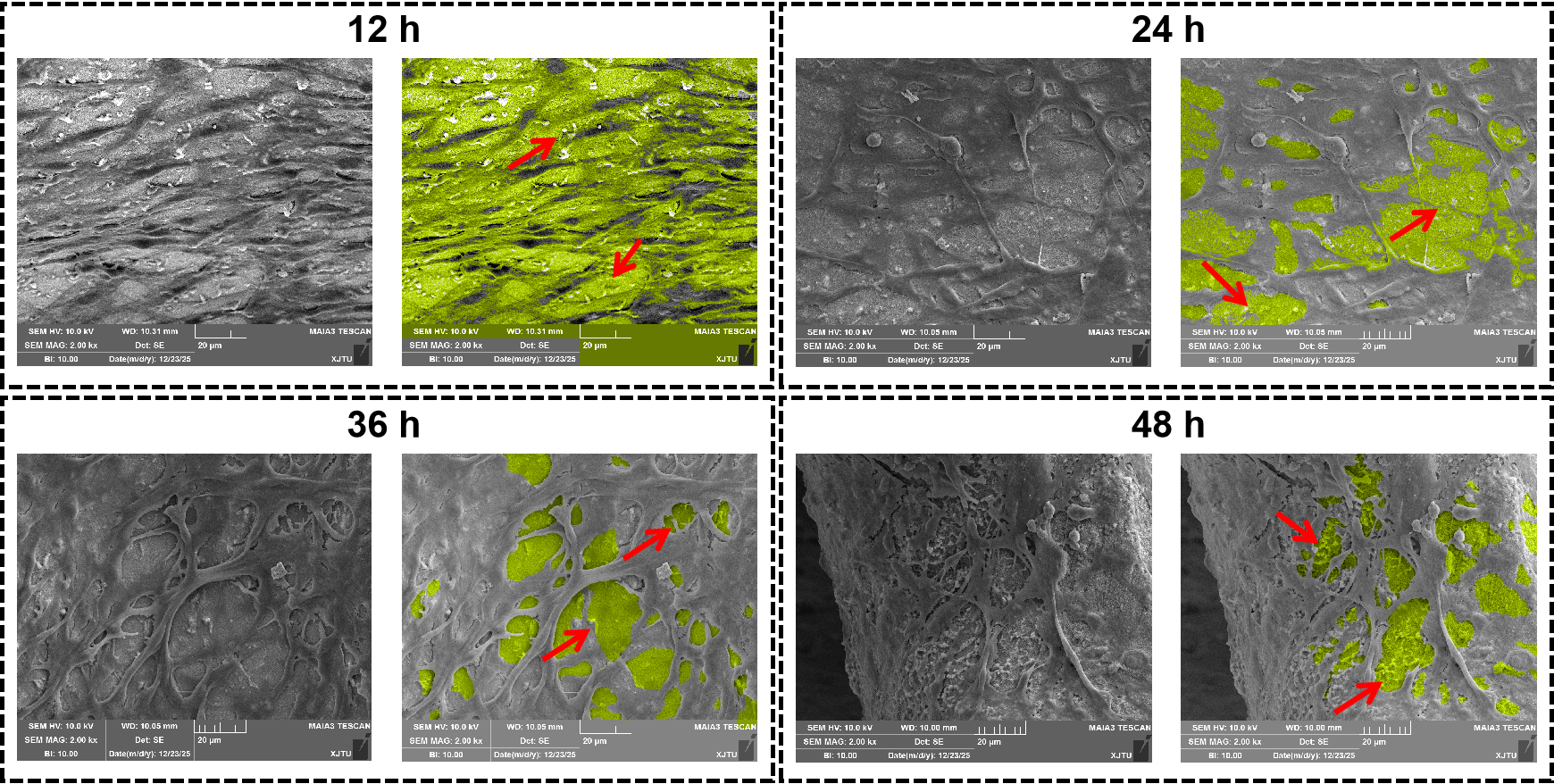


**Fig. S8.** High-magnification SEM revealed nanoscale tubular and fibrous features (tens to hundreds of nanometers), forming an interconnected network at the cell–substrate interface. These nanostructures are consistent with LDH-derived architectures, likely remodeled during early BMSC adhesion. The yellow regions represent pseudocolored areas of the Mg alloy scaffold surface not covered by BMSCs, while the red arrows indicate the nanostructures.


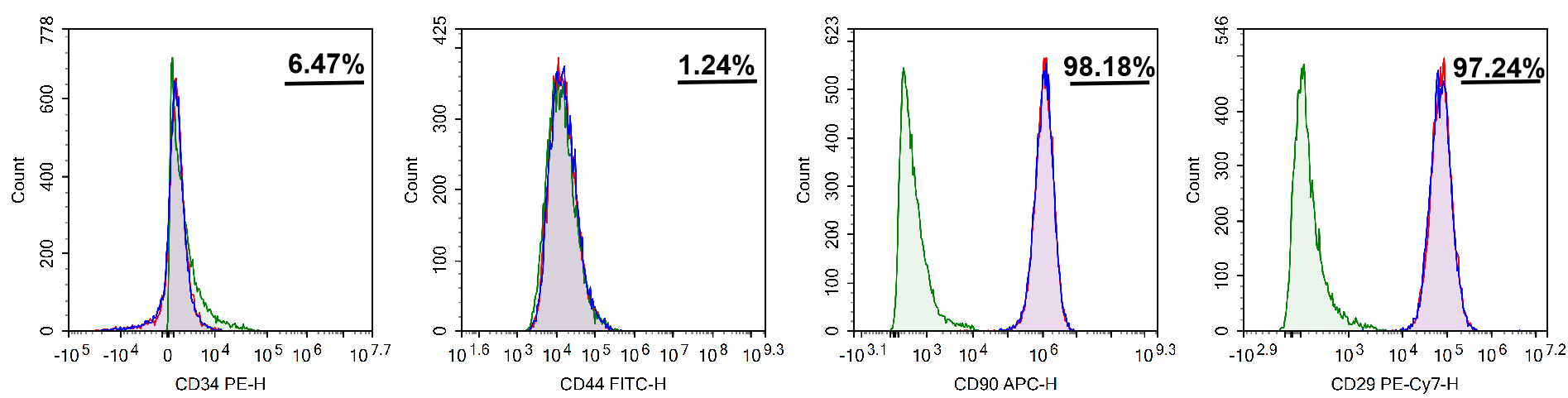


**Fig. S9.** **Flow cytometric analysis of surface antigen expression in different groups.** The expression levels of CD34, CD44, CD90, and CD29 were evaluated by flow cytometry across three groups. The green bars represent the Blank group (no cells), the blue bars represent the Control group (BMSCs), and the red bars represent the Experimental group (Mg + BMSCs).


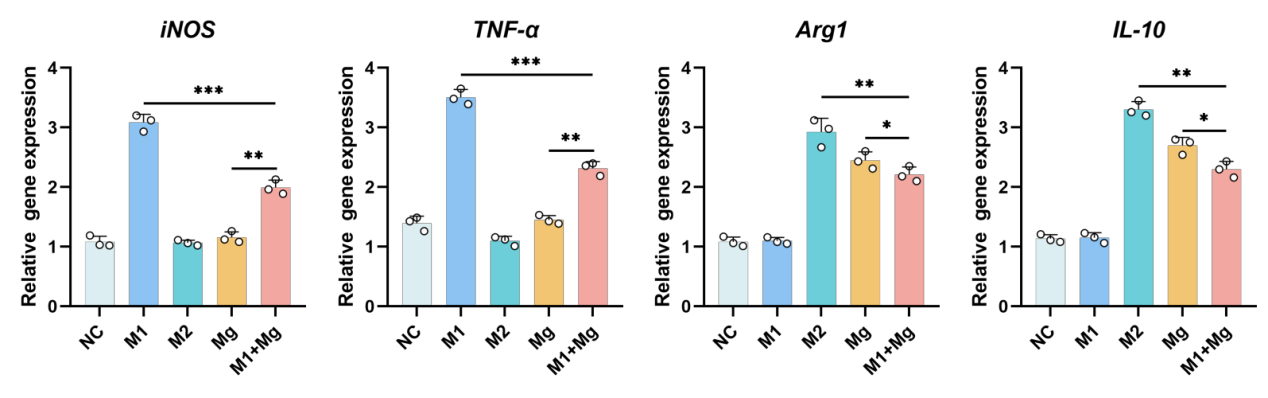


**Fig. S10.** Quantitative analysis of relative gene expression of iNOS, TNF-α, Arg1, and IL-10. Data are presented as mean values ± s.d. (n = 3). *p < 0.05, **p < 0.01, ***p < 0.001 (one-way ANOVA).


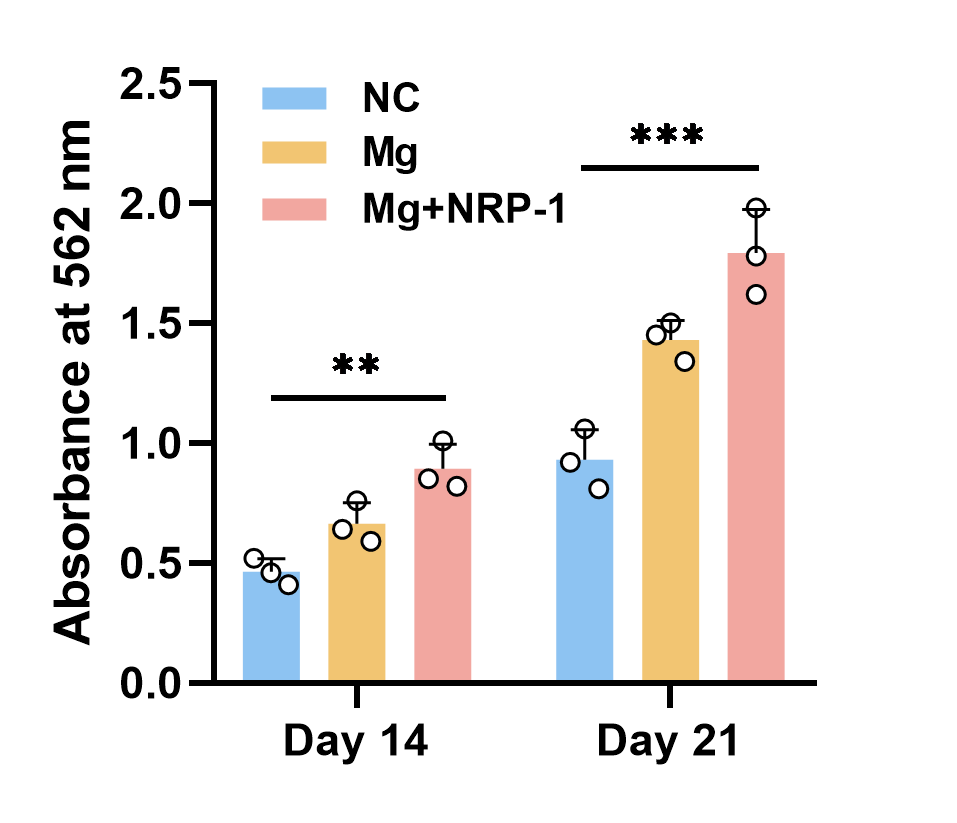


**Fig. S11.** Quantitative analysis of ARS staining was performed by CPC extraction, and mineralization was evaluated by measuring the absorbance at 562 nm.


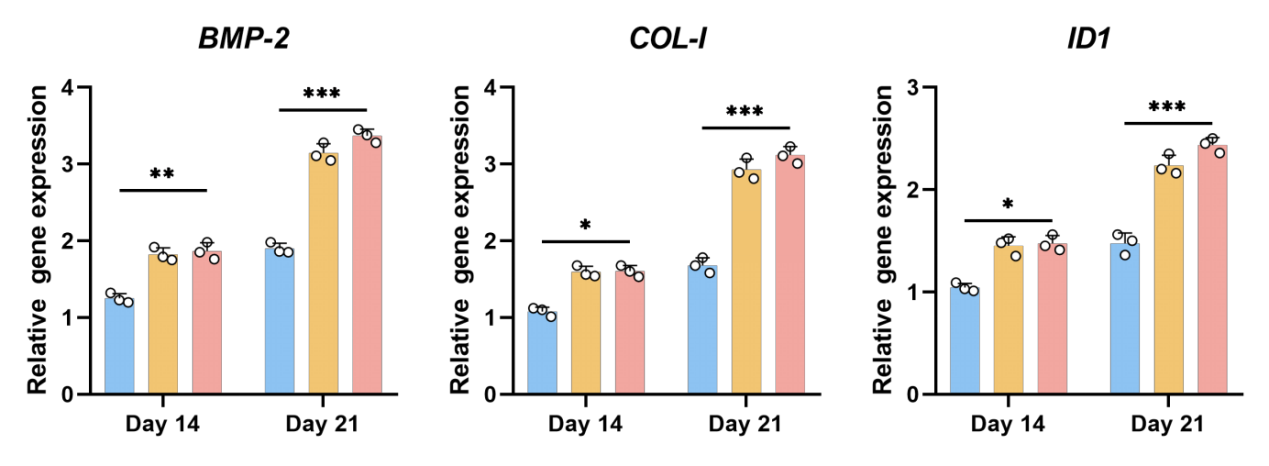


**Fig. S12.** Quantitative analysis of relative gene expression of BMP-2, COL-I, and ID1. Data are presented as mean values ± s.d. (n = 3). *p < 0.05, **p < 0.01, ***p < 0.001 (one-way ANOVA).


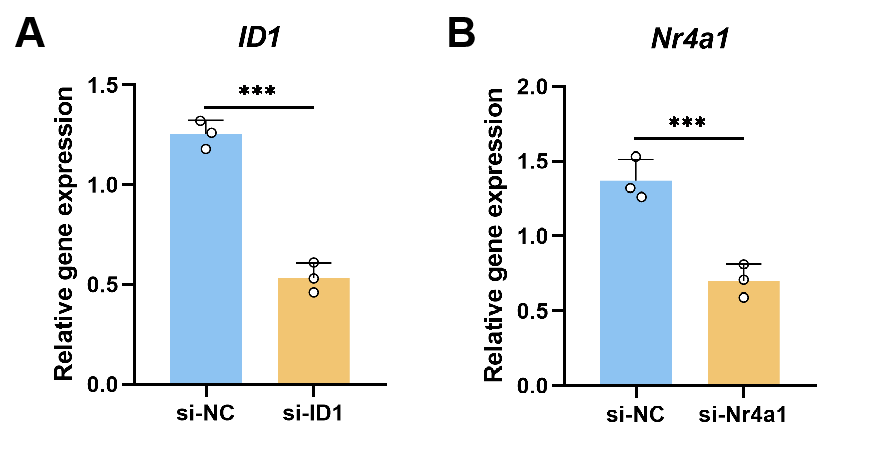


**Fig. S13. qRT-PCR validation of siRNA-mediated knockdown efficiency of ID1 and NR4A1.** Relative mRNA expression levels of **(A) ID1** and **(B) NR4A1** in BMSCs following transfection with gene-specific siRNAs (si-ID1 or si-NR4A1) or non-targeting scrambled siRNA (si-NC). Data are presented as mean values ± s.d. (n = 3). *p < 0.05, **p < 0.01, ***p < 0.001 (one-way ANOVA).


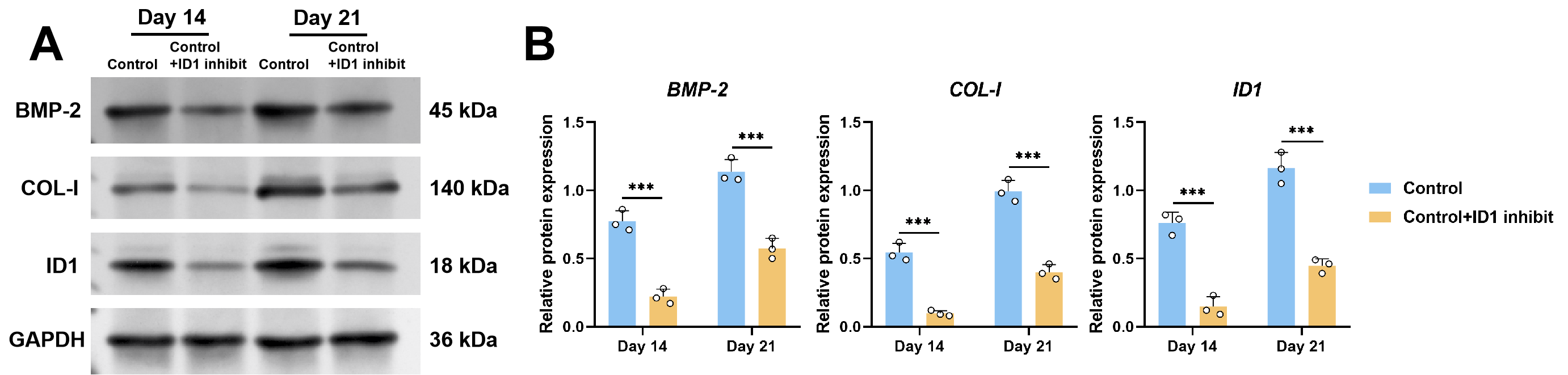


**Fig. S14.** (**A**) Western-blot analysis of BMP-2, COL-I, and ID1. (**B**) Quantitative analysis of relative protein expression of BMP-2, COL-I, and ID1. Data are presented as mean values ± s.d. (n = 3). *p < 0.05, **p < 0.01, ***p < 0.001 (one-way ANOVA).


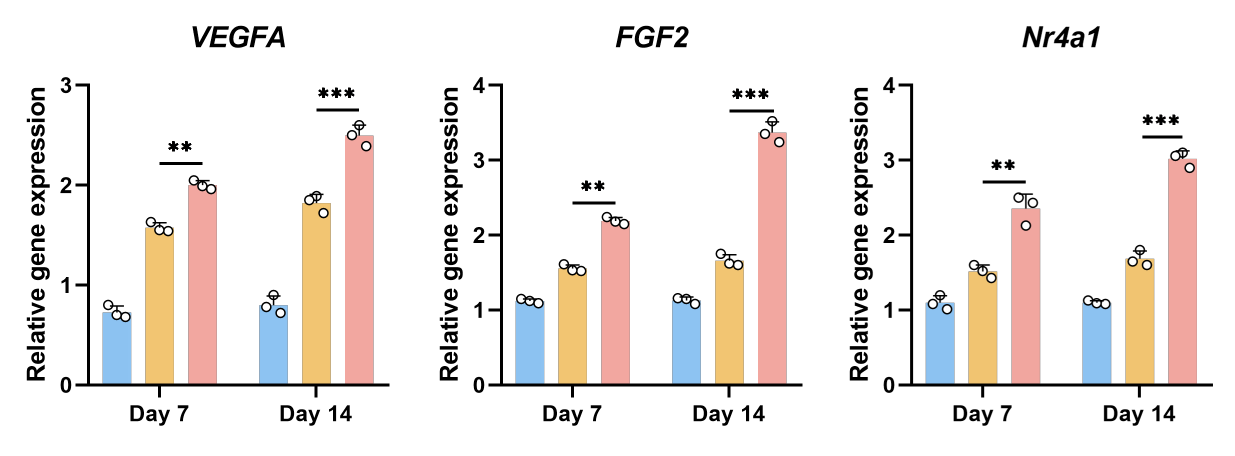


**Fig. S15.** Quantitative analysis of relative gene expression of VEGFA, FGF2, and Nr4a1. Data are presented as mean values ± s.d. (n = 3). *p < 0.05, **p < 0.01, ***p < 0.001 (one-way ANOVA).


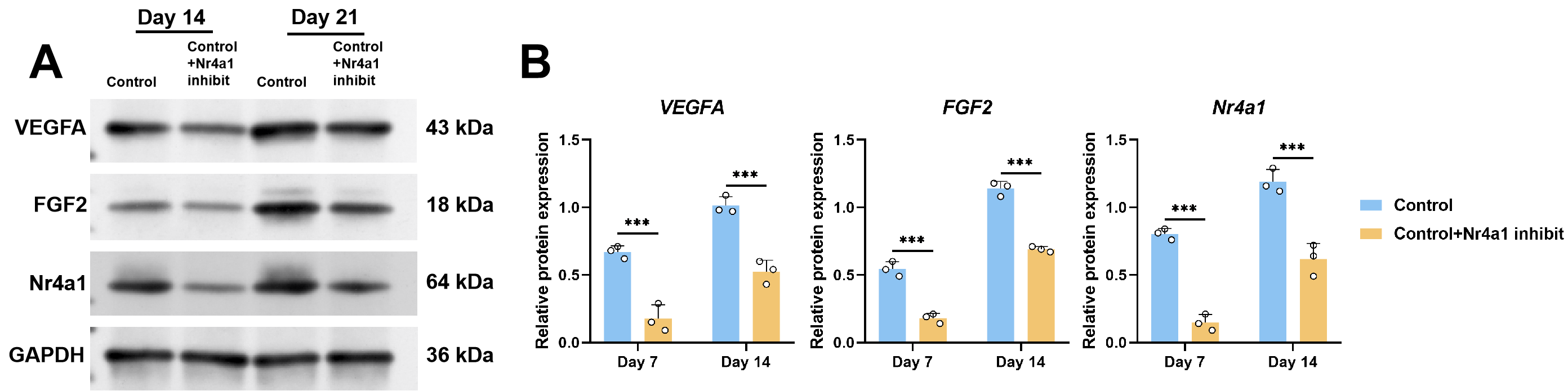


**Fig. S16.** (**A**) Western-blot analysis of VEGFA, FGF2, and Nr4a1. (**B**) Quantitative analysis of relative protein expression of VEGFA, FGF2, and Nr4a1. Data are presented as mean values ± s.d. (n = 3). *p < 0.05, **p < 0.01, ***p < 0.001 (one-way ANOVA).


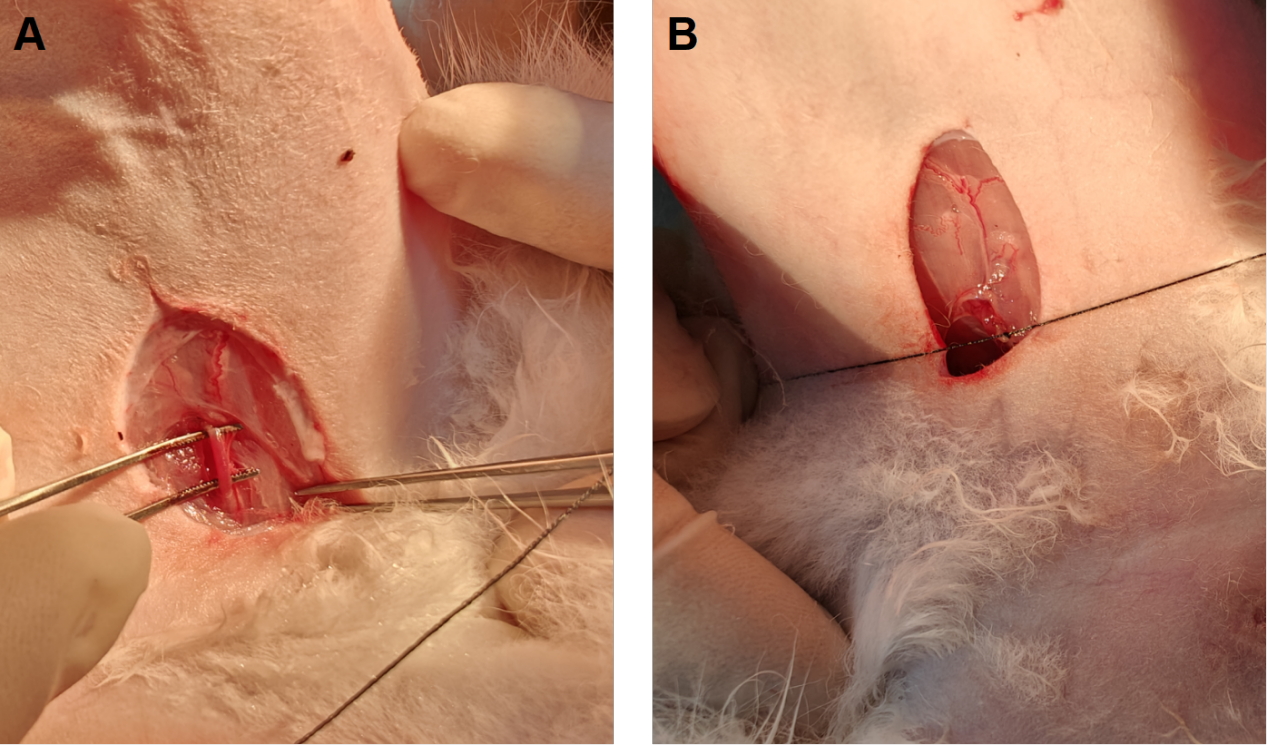


**Fig. S17.** Surgical procedure for femoral artery ligation in New Zealand rabbits. (**A**) The femoral artery was carefully exposed and isolated over a length of approximately 1.5 cm. (**B**) Both the proximal and distal ends of the isolated arterial segment were ligated to induce ischemia.


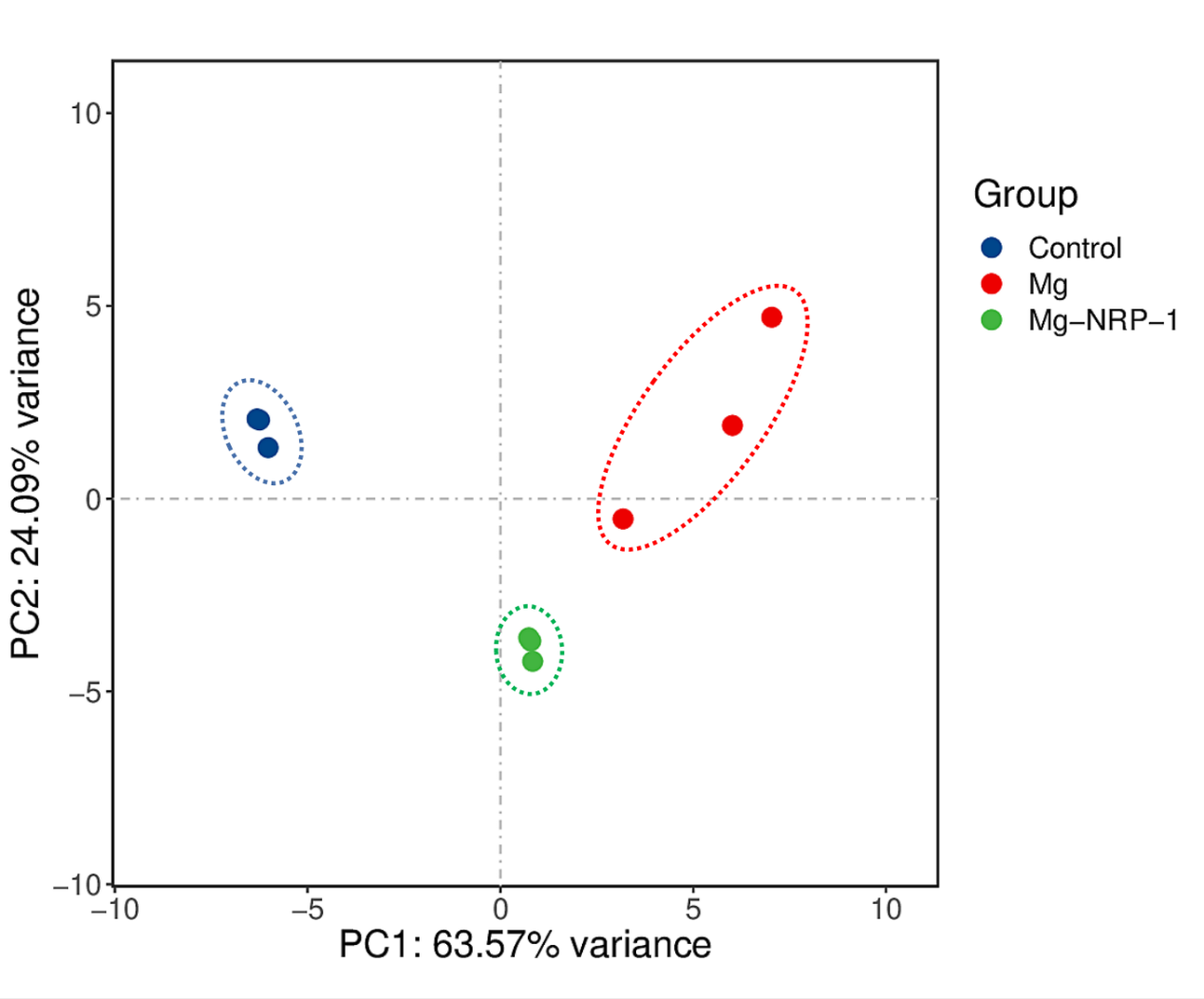


**Fig. S18.** PCA of RNA sequencing results.


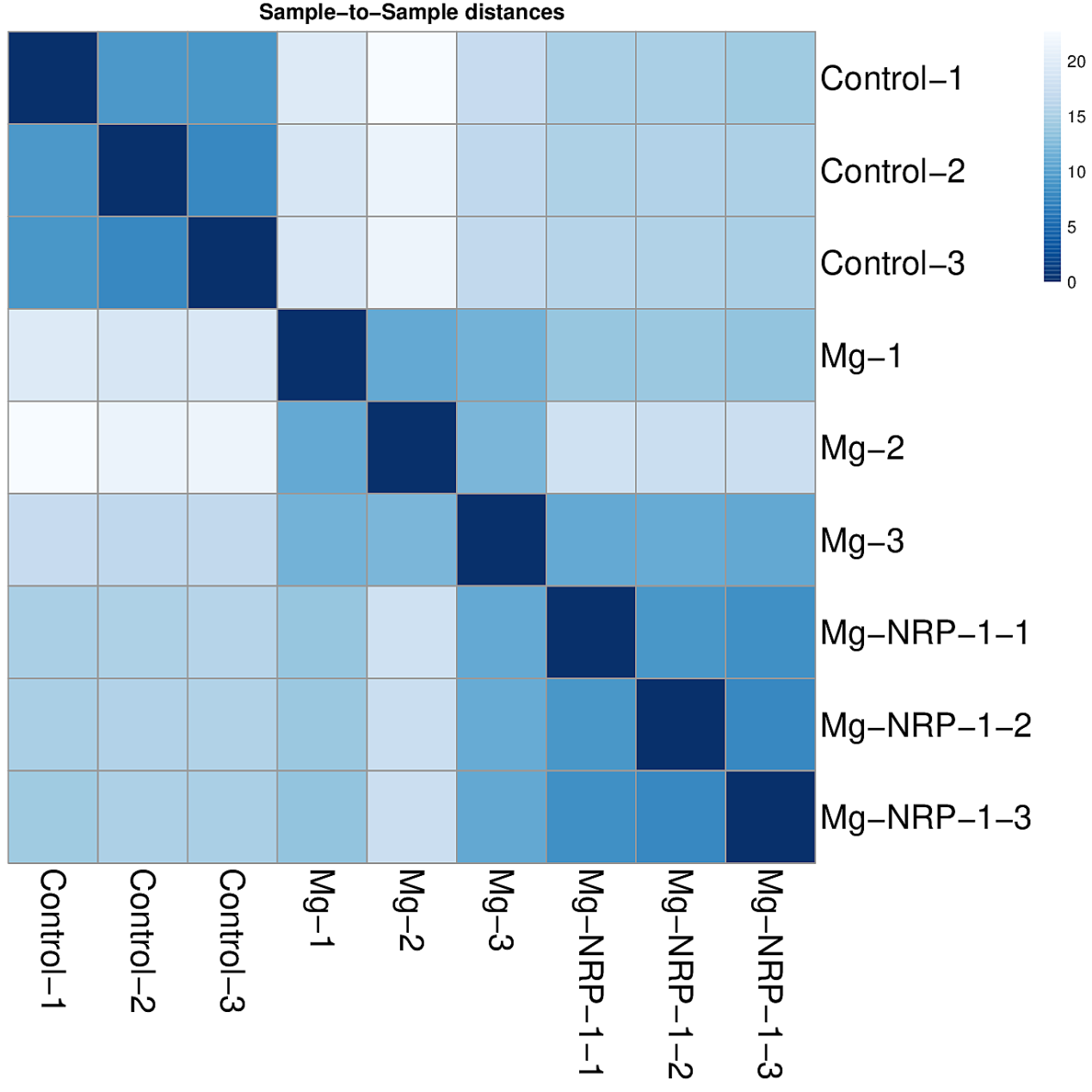


**Fig. S19.** RNA Seq correlation analysis of RNA sequencing results.


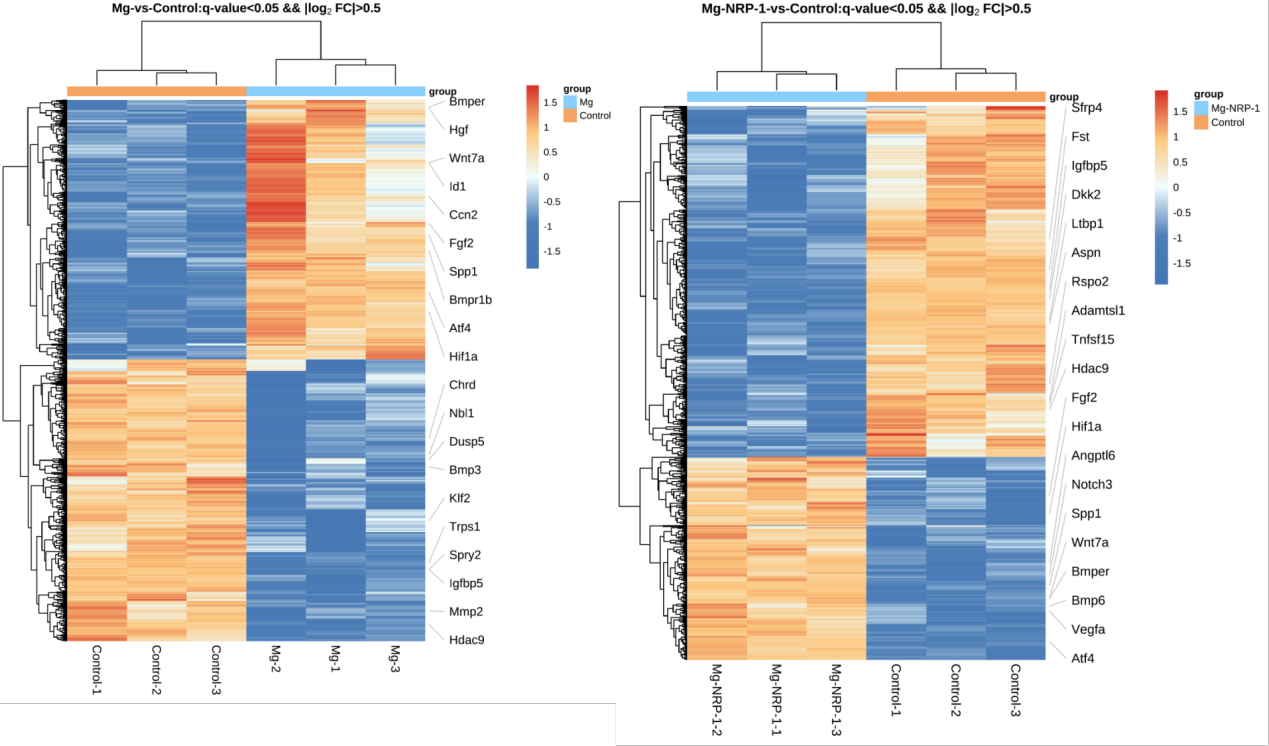


**Fig. S20.** Cluster analysis of differentially expressed genes of RNA sequencing results.


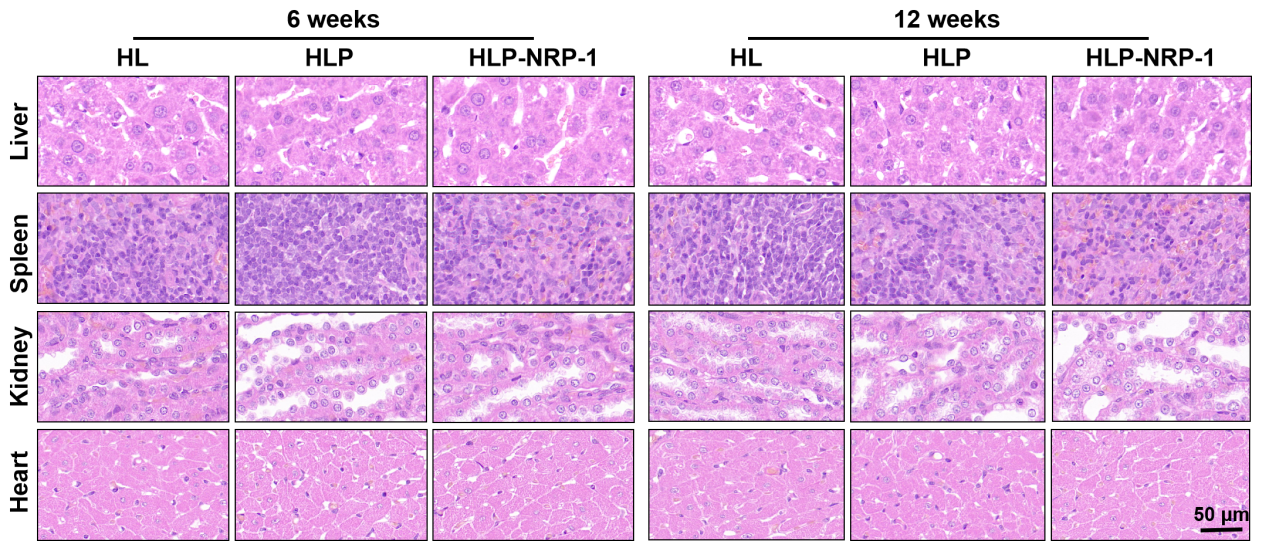


**Fig. S21.** Systemic toxicity analysis. H&E staining of liver, spleen, kidney and heart.
